# Advances and Applications of Metal‐Organic Frameworks (MOFs) in Emerging Technologies: A Comprehensive Review

**DOI:** 10.1002/gch2.202300244

**Published:** 2023-12-30

**Authors:** Dongxiao Li, Anurag Yadav, Hong Zhou, Kaustav Roy, Pounraj Thanasekaran, Chengkuo Lee

**Affiliations:** ^1^ Department of Electrical and Computer Engineering National University of Singapore Singapore 117583 Singapore; ^2^ Center for Intelligent Sensors and MEMS National University of Singapore Singapore 117608 Singapore; ^3^ Department of Chemistry Pondicherry University Puducherry 605014 India

**Keywords:** implantable electronics, metal‐organic frameworks, mid‐infrared nanoantennas, synthetic approaches, sensors

## Abstract

Metal‐organic frameworks (MOFs) that are the wonder material of the 21st century consist of metal ions/clusters coordinated to organic ligands to form one‐ or more‐dimensional porous structures with unprecedented chemical and structural tunability, exceptional thermal stability, ultrahigh porosity, and a large surface area, making them an ideal candidate for numerous potential applications. In this work, the recent progress in the design and synthetic approaches of MOFs and explore their potential applications in the fields of gas storage and separation, catalysis, magnetism, drug delivery, chemical/biosensing, supercapacitors, rechargeable batteries and self‐powered wearable sensors based on piezoelectric and triboelectric nanogenerators are summarized. Lastly, this work identifies present challenges and outlines future opportunities in this field, which can provide valuable references.

## Graphical abstract

Metal–organic frameworks (MOFs), hailed as the wonder material of the 21st century, exhibit unprecedented tunability, thermal stability, porosity, and surface area. This work highlights advancements in MOFs design and synthesis, delving into their applications in gas storage, catalysis, magnetism, nanomedical, sensing, light‐emitting diodes, and self‐powered wearable sensors via nanogenerators. The work also addresses current challenges, offering insights into future opportunities in this dynamic field.

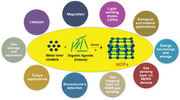

## 1 General Background

Metal‐organic frameworks (MOFs), also known as coordination polymers (PCPs), that are a fascinating class of porous crystalline materials have gained significant attention in a plethora of analytical and bioanalytical applications. These materials are constructed from metal ions/clusters and multidentate organic linkers via coordination bonds, which allow for great synthetic design, and properties such as large surface area, porosity, morphology, controllable shape, high stability, and multifunctionalities.^[^ ^1^, ^2^, ^3^ ^]^ Given these unique features, MOFs have been widely exploited in applications such as, but not limited to gas storage and separation,^[^ ^4^, ^5^ ^]^ photoluminescence,^[^ ^6^ ^]^ sensing,^[^ ^7^, ^8^ ^]^ magnetism,^[^ ^9^, ^10^ ^]^ catalysis,^[^ ^11^, ^12^ ^]^ optoelectronics,^[^ ^13^, ^14^ ^]^ drug delivery,^[^ ^15^, ^16^ ^]^ bioimaging,^[^ ^17^, ^18^ ^]^ conductivity,^[^ ^19^, ^20^ ^]^ energy storage,^[^ ^21^, ^22^ ^]^ etc. Recently, much more attention has been focused on exploring the applications of the gas sensing layer on micro‐electromechanical system (MEMS) devices, the gas trapping layer for Mid‐infrared (mid‐IR) gas sensing by the nanoantennae technology, and biomolecule detection in the microfluidic system^[^ ^23^, ^24^, ^25^, ^26^ ^]^ using a relatively new and unique class of MOFs. In addition, MOFs have been proven to be ideal sacrificial templates for fabricating nanoporous carbon, nanostructured metal oxides, and carbon–metal hybrid materials by changing the thermal conditions, which can perform excellent characteristics in electrochemical storage and conversion.^[^ ^27^, ^28^ ^]^

MOFs have a long history, dating back to the early 1990s when researchers began to investigate the synthesis and characteristics of these unique materials. The discovery of MOFs is often credited to the work of Omar M. Yaghi, and his team, in 1995 and 1998 who synthesized the first MOF, Zn_4_O(BDC)_3_·(DMF)_8_(C_6_H_5_Cl) named MOF‐5, which is composed of tetrahedral [Zn_4_O]^6+^ clusters bridged by BDC ligands (BDC = 1,4‐benzenedicarboxylate) to form a 3D cubic network. This MOF has specific properties such as porosity, a high surface area of 6,500 m^2^ g^−1^, X‐ray single crystal structure determination, and gas sorption properties.^[^ ^29^, ^30^ ^]^ This pioneering work marked the beginning of a new era in the science field of porous materials. Several MOFs such as isoreticular metal‐organic frameworks (IRMOF‐1 to IRMOF‐16), were also synthesized having a MOF‐5‐like topology.^[^ ^31^ ^]^ This development has received a great deal of interest in MOFs to fine‐tune their certain properties such as pore size, particle dimensions, surface area, etc., toward a specific application by way of a judicious choice of linkers and metal precursors. This type of study was also extended to various other MOFs, such as ZIF (zeolitic imidazolate framework), ZMOF (zeolite‐like metal‐organic framework), MAF (metal azolate frameworks), MPF (metal peptide framework), and bioMOF (metal biomolecule framework).^[^ ^32^ ^]^ Later, researchers synthesized the MOFs and named them after the University names. Some of the examples are UiO‐66 (University of Oslo), HKUST (Hong Kong University of Science and Technology), MIL (Materials Institute Lavoisier), SNU (Seoul National University), JUC (Jilin University China), ITQMOF (Instituto de Tecnología Química metal‐organic framework), POST (Pohang University of Science and Technology) and, CUK (Cambridge University KRICT), etc. Wilmer et al. generated 137 953 hypothetical MOFs from a library of 102 building blocks and screened 300 hypothetical MOFs with a higher capacity for methane storage.^[^ ^33^ ^]^ Since then, an unlimited number of MOFs with different combinations of metal precursors and organic linkers have been developed to tune the desired properties such as gas storage and separation, magnetic, catalytic, electrical, and optical properties, etc. for a wide variety of applications.

The synthesis of MOFs can be influenced by several factors such as the nature of metal ions, typical coordinating organic linkers (cyanide, pyridyl, carboxylate, azolates, sulfonates, phosphonates, hydroxyl), solvent system, temperature, and counter ions.^[^ ^3^, ^34^, ^35^, ^36^ ^]^ By employing the length or functionalities of the organic linker and preferred coordination geometries of the metal center (octahedral, tetrahedral, square planar, etc.), pore size as well as the shape of the pore can be controlled. Multi‐topic organic ligands that have a number of coordinating functional groups with diverse coordination modes usually generate 2D or 3D structures upon coordinating with metal centers. Especially, first‐row transition metal ions, alkaline earth metal ions, and lanthanides have been mostly used for the production of MOFs because of their wide range of coordination numbers, oxidation states, and geometries. Several methods have been used to achieve the stability of MOFs using high valent metal cations such as Al^3+^, Cr^3+^, Fe^3+^, Ti^4+^, and Zr^4+^ ions and carboxylate linkers through metal–polyazolate interactions or creating hydrophobic structures. A range of non‐covalent forces such as π–π stacking, hydrogen bonding, and van der Waals interactions have been employed not only to stabilize the MOF structures but also to play a major role in the accommodation of various guest molecules. Generally, several methods such as hydrothermal or solvothermal synthesis, ionothermal, mechanochemical, slow diffusion, ultrasonic, microwave, and electrochemical, have been conveniently employed in the production of robust and highly ordered metal‐organic frameworks. As a kind of multifunctional material, MOFs have aroused great interest by presenting numerous features such as high surface areas (ranging from 1000 to 10 000 m^2^ g^−1^) and customizable pore sizes/characteristics (14–98 Å) with a low framework density (e.g., 0.124 g cm^−3^), high chemical and thermal stabilities, high luminescence, making them a potential candidate for applications including gas storage and separation, catalysis, magnetism, conductivity, drug delivery, and sensing applications so on.^[^ ^4^, ^5^, ^6^, ^7^, ^8^, ^9^, ^10^, ^11^, ^12^, ^13^, ^14^, ^15^, ^16^, ^17^, ^18^, ^19^, ^20^, ^21^, ^22^, ^37^, ^38^, ^39^, ^40^, ^41^, ^42^, ^43^, ^44^, ^45^, ^46^, ^47^, ^48^ ^]^ In addition, several research teams examined MOFs and their composite materials as catalysts for the electrocatalytic oxygen reduction reaction (ORR), hydrogen evolution reaction (HER), and oxygen evolution reaction (OER).^[^ ^43^, ^49^ ^]^

Apart from these applications, a fascinating and remarkable development in this century is that MOFs can be used as light/energy harvesting materials through triboelectric nanogenerators (TENG),^[^ ^50^ ^]^ which can convert mechanical energy into electricity based on the combination of electrostatic induction and contact electrification in order to drive small electronics and make self‐powered electronics networks viable, functioning as active sensors for medical, human‐machine, environmental‐monitoring and security.^[^ ^51^ ^]^ As examples of the utility of MOFs toward TENG technology, a family of zeolitic imidazole frameworks, HKUST‐1, etc., have elegantly been highlighted.^[^ ^52^, ^53^, ^54^ ^]^ From the literature analysis, it can be understood that depending on the functional group, MOFs displayed positive or negative triboelectric effects. For instance, fluorine‐functionalized MOFs exhibited a high tribonegative nature due to the enhanced charge transport capability.^[^ ^55^ ^]^ The practical implementation of MIL‐TENG in several low‐rating electronics confirmed the utilization of this developing material in future biodegradable implanted medical devices.^[^ ^56^ ^]^

**Figure 1:**
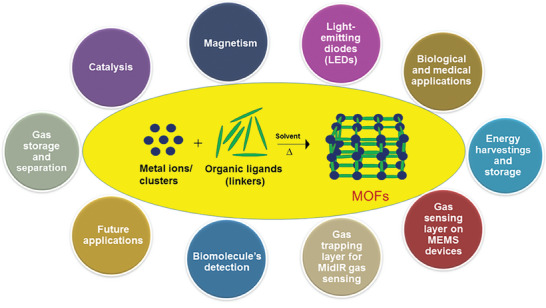
Synthesis and applications of MOFs.

By imparting exclusive knowledge on constructing MOFs from various metal ions and organic linkers and precisely controlling weak interactions in the confined pores, researchers can develop multifaceted approaches to impart new functionalities, which promote their use in industries. Considering the rapid development in this area, this review briefly outlines the general aspects of the synthesis and process of MOFs and derivatives for multidisciplinary applications. First, we discuss the general introduction and preparation strategies of MOFs. Subsequently, the unique attributes of MOFs and their extensive applications are discussed (**Figure** **1**). Finally, we will end our review with a summary and future trends toward MOF‐based technologies. The purpose of this review is to arouse tremendous research attention in the preparation and fabrication of MOFs via different synthesis techniques, advantages, and challenges and promote the development of these MOFs in advanced multidisciplinary applications. We hope that the present review will serve as a guide to explore the value of MOFs from both academic and industrial perspectives.

## 2 MOFs Preparation and Fabrication

For the past two decades, various synthetic methods have been utilized for the production of new MOFs with different crystal structures, high surface area, particle size, pore size distribution, and morphologies, providing researchers with a unique platform for modifying desirable properties.

### 2.1 Hydrothermal Synthesis

**Figure 2:**
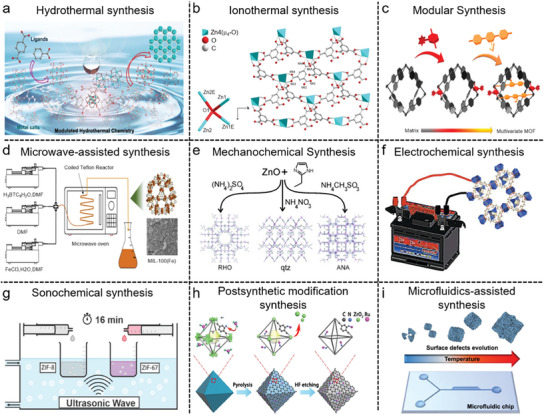
MOF preparation and fabrication a) Hydrothermal Synthesis of MOFs. Reproduced with permission.^[^ ^60^ ^]^ Copyright 2022, American Chemical Society. b) Ionothermal synthesis of MOFs. Reproduced with permission.^[^ ^67^ ^]^ Copyright 2008, Elsevier. c) Modular Synthesis of MOFs. Reproduced with permission.^[^ ^70^ ^]^ Copyright 2019, John Wiley and Son. d) Microwave‐assisted synthesis of MOFs. Reproduced with permission.^[^ ^72^ ^]^ Copyright 2020, Elsevier. e) Mechanochemical Synthesis of MOFs. Reproduced with permission.^[^ ^76^ ^]^ Copyright 2010, John Wiley and Son. f) Electrochemical synthesis of MOFs. Reproduced with permission.^[^ ^81^ ^]^ Copyright 2012, American Chemical Society. g) Sonochemical synthesis of MOFs. Reproduced with permission.^[^ ^89^ ^]^ Copyright 2020, Elsevier. h) Postsynthetic modification synthesis of MOFs. Reproduced with permission.^[^ ^90^ ^]^ Copyright 2017, American Chemical Society. i) Microfluidics‐assisted synthesis of MOFs. Reproduced with permission.^[^ ^96^ ^]^ Copyright 2022, Elsevier.

Hydrothermal method is one of the synthesis techniques used to produce single crystals of MOFs by the combination of organic ligands with metal salts in a water medium. Since water is used as a solvent, this method is termed a hydrothermal method. An autoclave (a steel pressure vessel) lined by Teflon is used to supply nutrients via water to promote crystal formation as a result of a temperature difference within the growth chamber.^[^ ^57^ ^]^ Compound crystallization is possible at high vapor pressures and temperatures thanks to hydrothermal synthesis. The growth of a single crystal is caused by the solute nourishment, which dissolves at the hotter end and gets deposited at the cooler end. Under this condition, the temperature of the solvent increases beyond its boiling point and atmospheric pressure but reduces its dielectric constant and viscosity, which results in an enhanced diffusion process followed by crystal growth. In an autoclave walled with Teflon, this procedure is normally performed under pressure and at temperatures below 200 °C. Through this technique, not only MOFs but also polyoxometalates (POMs), extended metal oxides, and covalent organic frameworks (COFs) have been prepared.^[^ ^58^ ^]^ MOFs having high vapor pressure close to their melting points through *in situ* ligand synthesis can also be prepared.^[^ ^59^ ^]^ The bridging ligand used in the preparation of MOFs remains intact within the motif during the entire synthesis process, which is different from zeolites (**Figure** **2**a).^[^ ^60^ ^]^

The advantages of this hydrothermal synthesis are simple and safe and has been used in the synthesis of high‐performance MOFs and their composites with controllable size and structure. The reagents may experience unpredicted chemical transformations to afford new ligands in situ during this process. The disadvantages of this technique are the use of costly autoclaves, high temperature, and high pressure, impossibility of observing the growth of crystals, longer synthesis time, etc.^[^ ^61^ ^]^

### 2.2 Solvothermal Synthesis and Ionothermal Synthesis

The solvothermal method is also a time‐consuming synthesis technique (growth of crystal occurs within a time period of hours to days) primarily used for the production of MOFs but using the minimum quantity of solvents. In this technique, crystalline MOFs are produced through a cooperative process between organic ligands and isolated metal ions, which aid in the controlled creation of MOFs like the hydrothermal technique. However, the primary distinction in solvothermal synthesis is the nature of the precursor solution, which is often not aqueous. This method combines the advantages of both hydrothermal and sol‐gel methods, thus allowing fine‐tuning of the experimental parameters to control the MOFs' size, shape, and crystallinity^[^ ^57^, ^62^ ^]^ by varying temperature, solvent, precursor, and duration of the reaction. In order to produce MOF crystals of excellent quality, it is imperative to the selection of solvent, reactants' solubility, and how they interact.^[^ ^63^ ^]^ The length of the reaction, the temperature of the reaction, the rate of stirring, and the ratio of metal/ligand all have an impact on the morphology of the resultant MOFs. Using particular reactants and solvents, solvothermal synthesis has demonstrated encouraging results in the synthesis of various MOFs, including ZIF‐67 and ZIF‐8.^[^ ^63^, ^64^ ^]^ However, some experimentation and trial‐and‐error studies are still required to dictate the suitable conditions for growing crystalline frameworks.^[^ ^65^ ^]^ The synthesized MOFs can be used in a variety of industries because they provide control over the stability, surface area, porosity, and functionality of the materials. Especially, this technique is very useful for the synthesis of titanium dioxide, graphene, carbon, and other nanostructured materials. The advantages of this solvothermal synthesis are simple and safe, easy operation, crystallinity, and excellent yield of products. New ligands formed in situ may generate new types of MOF structures, which are not performed under mild reaction conditions when the reagents undergo an unprecedented chemical transformation in this process. The costly autoclaves, observation of growing crystal, time‐consuming that can take days or weeks, and the use of hazardous solvents in the reaction mixture are the major issues of this technique.^[^ ^61^, ^66^ ^]^

Ionothermal synthesis has become a viable alternative to solvothermal synthesis in order to get around some of its drawbacks, such as problems with inorganic precursor solubility and hydrogen bond interference when using water as a solvent. The ionothermal approach efficiently dissolves the precursors and promotes the synthesis of desired MOFs with enhanced properties by using a low‐volatility ionic liquid as both the environment‐friendly solvent and the structure‐directing agent. For example, Xu and coworkers prepared zinc trimesate ([Zn_4_(BTC)_2_(µ_4_‐O)(H_2_O)_2_]) with the help of Zn(NO_3_)_2_.6H_2_O and trimesic acid using 1‐ethyl‐3‐methylimidazolium bromide ionic liquid as a solvent by ionothermal method (Figure 2b).^[^ ^67^ ^]^ Due to peculiar features such as low vapor pressure, high solubility for organic molecules, high thermal stability, wider liquid range, and nonflammability, the usage of ionic liquids as an environmentally‐friendly reagent for the synthesis of MOFs and other classes of materials such as zeolites and chalcogenides is promising. In addition, this method has promised to enable us to effectively produce a variety of reaction environments and thus MOFs through the fine selection of cations and anions of ionic liquids as counterions and/or as templates. The disadvantages of this method include the inseparable cations that are residing in the pores of MOFs from the solvents.^[^ ^68^ ^]^

### 2.3 Room Temperature Synthesis or Modular Synthesis

This process belongs to a unique class of solvothermal methods where heating is not necessary for the production or crystallization of MOFs from the reaction of organic ligands with isolated metal ions. The solvent used in the reaction mixture is kept at room temperature to crystallize MOFs under mild conditions. During the course of reaction, bases such as triethylamine are used, which results in the deprotonation of the organic ligands, leading to the formation of MOF as a precipitate.^[^ ^69^ ^]^ This method is usually a preferred route for the preparation of MOFs owing to the easy and simple operation, and good yield without supplying extra energy. It is a time‐consuming process within period of hours to days, and some of the reactions need to be performed under hazardous conditions using DMF (solvent), hydrofluoric acid (acid modulator), etc (Figure 2c).^[^ ^70^ ^]^

### 2.4 Microwave (MW)‐Assisted Synthesis

One of the most promising alternatives to conventional methods is microwave (MW) irradiation, which has been widely utilized in the synthesis of MOFs due to its numerous advantages. One of the main advantages of this technique is the quick heating time along with the energy‐saving process, higher yield, and high purity, making it more energy and time‐efficient than traditional methods. By using electromagnetic waves at the right frequency, it is possible to get molecules in a solution to collide, which increases their kinetic energy and boosts the temperature of the entire system.^[^ ^71^ ^]^ The effectiveness of the heating process is increased by the radiation's direct interaction with the solution. As a result, microwave synthesis promotes a high nucleation rate and quick crystallization. Microwave synthesis makes it possible to produce MOFs in a matter of minutes as opposed to conventional solvothermal techniques, which demand lengthy annealing durations. For example, Kim et al. reported a scalable MOF synthesis route based on microwave volume heating in a continuous‐flow tubular reactor (Figure 2d). The system achieved continuous crystallization of MIL‐100(Fe) under relatively mild conditions (temperature: 100–110 °C, time: 50 min), with a space‐time yield as high as ≈771.6 kg m^−3^ per day.^[^ ^72^ ^]^ In a comparative study by Choi and coworkers,^[^ ^73^ ^]^ the structural and surface properties of MOF‐5 were investigated using microwave heating and solvothermal methods. microwave heating at different power levels, irradiation times, temperatures, solvent concentrations, and substrate compositions yielded uniform cubic MOF‐5 crystals within 30 min, with an average size of 20–25 µm and a surface area of 3008 m^2^ g^−1^. In contrast, the conventional solvothermal method required 24 h at 105 °C to synthesize MOF‐5 crystals with a size range of 400–500 µm and a surface area of 3200 m^2^ g^−1^. MOFs were successfully created by Ni and Masel utilizing microwave synthesis. These MOFs were produced in a microwave synthesizer at 150 W for only 25 s, as opposed to their usual solvothermal synthesis requirements of 120 °C for 21 h.^[^ ^74^ ^]^ The microwave approach has an effect on crystal size and shape and works greatly for producing MOFs on a big scale as well. Using a MW‐assisted solvothermal technique, Bag and colleagues created isostructural microporous lanthanide MOFs in ≈5 min.^[^ ^75^ ^]^ In contrast, the solvothermal approach needed a lengthy 2‐day reaction time and an extra 5‐day evaporation period. Apart from quick crystallization, microwave‐assisted synthesis shows additional advantages such as phase selectivity, reduction of particle size, and morphological controls while it lacks the large crystal formation to get good structural data.^[^ ^66^ ^]^

### 2.5 Mechanochemical Synthesis

MOF creation by mechanochemical synthesis offers a lot of potential to be both environmentally friendly and economically advantageous (Figure 2e).^[^ ^76^ ^]^ In this process, the intermolecular bonds experienced mechanical breakdown followed by chemical modification after generating new bonds when the metal salts and organic ligands were ground in a mechanical ball milling machine. A single‐phased highly crystalline MOFs having guest molecules in their voids have been formed. This approach has the benefits of not using solvents, producing safe by‐products, quick reaction times, and giving a high yield of products at room temperature. However, the addition of a minimum quantity of solvents stimulates the mechanochemical reactions by increasing the mobility of the reactants at the molecular level. HKUST‐1 was synthesized by the reaction of copper acetate and trimesic acid for 25 min, and it was demonstrated that the residual acetic acid molecules blocked the pores and caused mesopores to develop. The high surface area of 1713 m^2^ g^−1^ was produced by performing a single post‐synthesis activation to remove the gaseous by‐product.^[^ ^77^ ^]^ This eco‐friendly method is attractive to prepare a variety of MOFs in a short reaction time at room temperature without using solvents, high temperature, or high pressure which are essential in the hydro/solvo thermal method. The disadvantages of this method are the difficulty of the precise control of synthesis, requirement of special grinders/mills, large consumption of energy, low work efficiency, possible impurities from milling reactors, etc.^[^ ^78^, ^79^, ^80^ ^]^

### 2.6 Electrochemical Synthesis

MOFs have been prepared using the electrochemical technique, which provides a quick synthesis and gentle reaction conditions. This method helps to keep unwanted anions such as halides, perchlorate, or nitrate from entering large‐scale industrial operations. Instead of using metal salts, metal ions are employed, which are supplied through anodic dissolution as a metal source. This high‐reactive metal species is then added by anodic dissolution to the reaction mixture of an organic ligand and an electrolyte. To avoid the deposition of metals on the cathode, protic solvents are often used but H_2_ is generated in this process (Figure 2f). The benefit of this technique is faster synthesis at lower temperatures without anionic residues.^[^ ^81^ ^]^ The structure and shape of the produced MOFs are significantly influenced by the reaction time and the solvent content as well. If given enough time, a dense crystal layer may form partially, while the supporting mesh may suffer structural damage. For example, low temperatures (50 °C) and quick reaction times made the electrochemical approach ideal for producing HKUST‐1 (CuBTC). The porous copper support is outgrown by the resulting crystal layers.^[^ ^82^ ^]^ Another example of MOF, CuTATB (H_3_TATB = 4,4′,4′′‐s‐triazine‐2,4,6‐triyl‐tribenzoic acid) was synthesized as a bulk powder by Sachdeva and co‐workers.^[^ ^83^ ^]^ The bulk powder contained agglomerated particles with a BET surface area of 570 m^2^ g^−1^ and showed needle‐like features. Smaller particles developed as a result of a quick nucleation process when CuTATB was grown on the copper surface, which prevents interpenetration. The major disadvantages of this method are low productivity and unsuitable for large‐scale synthesis due to large‐size electrochemical equipment and large area metal electrodes.^[^ ^80^ ^]^

### 2.7 Sonochemical Synthesis

Sonochemical synthesis or Ultrasound‐assisted technique (20 kHz‐10 MHz) provides an alternative way of preparing MOF crystals in a rapid and environmentally friendly way. This technique can accelerate homogeneous nucleation along with the formation of smaller particle sizes in a substantial reduction in crystallization time compared with the solvothermal method. Upon interaction of high energy ultrasound with liquid, an acoustic cavitation (the process of creation, growth, and collapse of a bubble under altering pressure) occurs followed by produces of high temperatures of 5000–25000 K and pressures as well as high heating and cooling rate. Literature studies have shown that MOFs can be produced cost‐effectively at high yields via this technique.^[^ ^84^ ^]^ Qiu and coworkers first reported sonochemical synthesis of a MOF [Zn_3_(BTC)_2_] (BTC = benzene tricarboxylicacid) in ethanol and studied its selective sensing toward organoamine.^[^ ^85^ ^]^ In another study, HKUST‐1 showed its behavior on the effect of reaction time on particle size during preparation of this method.^[^ ^86^ ^]^ Through this technique, Son and co‐workers reported good quality MOF‐5 crystals of 5–25 mm in size within a short period (30 min) in comparison with the solvothermal technique (24 h).^[^ ^87^ ^]^ This technique permits a low power consumption and is cheaper compared to a microwave process because the induction period is not required before stabilization of the sonicator, and the duration of synthesis is very short. In general, the size of the nanocrystals is smaller than that of the solvothermal method. For large‐scale production, an economical solvent, 1‐methyl‐2‐pyrrolidone (NMP) can be used for the production of MOFs instead of using DEF in the solvothermal synthesis. Khan and Jhung realized that during the synthesis of MIL‐53 by solvothermal, ultrasonic, and microwave techniques, the rate of crystallization and rate of crystal development were found in the order ultrasound (US) > microwave (MW) > solvothermal (ST).^[^ ^88^ ^]^ In addition, they discovered that microwave and ultrasound techniques performed well in homogenous nucleation, and the production of tiny crystals in a short period. Recently, Yao et al. developed a gentle and efficient ultrasound‐assisted synthesis method to prepare ZIF‐8, ZIF‐67, and Co/ZIF‐8 with well‐designed particle sizes and morphologies (Figure 2g). Tunable particle size from 35 nm to over 300 nm was achieved by adjusting the cobalt content doped into the heterometallic ZIF‐8 structure.^[^ ^89^ ^]^ This technique possesses a limited heating depth with a low product yield which impedes the application of this technique to large‐scale synthesis of MOFs.^[^ ^80^ ^]^

### 2.8 Postsynthetic Modification (PSM) Synthesis

Postsynthetic modification (PSM) became widely adopted to expand the scope and versatility of functional groups that are incorporated into parent MOFs (Figure 2h).^[^ ^90^ ^]^ Cohen described the concept of “postsynthetic modification” and performed organic reactions on MOFs to produce topologically identical but functionally diversified MOFs using PSM strategy via single or multistep reactions.^[^ ^91^ ^]^ To fabricate different functional groups containing MOFs, several PSM routes can be made on the metal exchange, ligand exchange, guest replacement, and metalation of open coordinate sites in ligands, which can work individually or synergistically.^[^ ^92^ ^]^ PSM could be accomplished only when the MOFs do not undergo degradation without affecting structure, crystallinity, and porosity during the reaction. It also permits to incorporate of chiral recognition sites to control chiral selectivity into MOFs.^[^ ^93^ ^]^ A team of Bennet found that the amine group presented in ZIF MOFs is not only responsible for lowering melting temperature but also permitting to tuning of surface properties from hydrophilic to hydrophobic in the presence of octyl isocyanate through the PSM method.^[^ ^94^ ^]^ This strategy is an efficient route to generate new MOFs under mild reaction conditions owing to its easy‐to‐manipulate, good universality, highly oriented decoration, and available heterogeneous membrane structure without significant interference. The drawbacks of this method include newly‐formed intracrystalline or intercrystalline defects and crystallinity deterioration.^[^ ^95^ ^]^

### 2.9 Microfluidics‐Assisted Synthesis

Microfluidics is a chemical synthesis method based on the control of fluid flows at micro‐ and nano‐scale using chips. This method permits the continuous production of MOFs by controlling accurate parameters in synthesis, representing a step toward intensification, versatility, and scalability in the use of MOFs (Figure 2i).^[^ ^96^ ^]^ Several experimental studies showed that this approach can be used for the synthesis of different MOFs including carboxylates‐based MOFs (HKUST‐1, Cu‐BTC, MOF‐5, IRMOF‐3, UiO‐66) and imidazolates‐based MOFs (ZIF‐7 and its derivatives).^[^ ^97^ ^]^ In a recent study, Mao and co‐workers^[^ ^98^ ^]^ demonstrated the encapsulation and extraction of DNA, which is an extraordinary data storage platform within MOFs in a short period of 10 and 5 min, respectively for automated and integrated data storage. DNA microlibrary@MOFs underwent not only enhanced stability but also could be able to read out fast retrieval of data after aging. The synthesis of MOFs using a conventional solvothermal approach takes 72 h, while the synthesis on a microfluidic chip takes ≈ 1 min. The use of microfluidic‐assisted synthesis has several advantages including morphological control, fine particle size, good reproducibility, lack of large work areas, and expensive equipment. This approach also offers the possibility of defect‐free MOF‐based hollow fiber membranes saving reactants in comparison with conventional approaches. Although this process showed several advantages over others, there are some issues while the preparation of MOFs undergoes at high temperatures. The disadvantages of this technique include the need for a large area, high wattage, costly, high‐maintenance equipment, and the release of waste or byproducts that are harmful to the environment.^[^ ^99^ ^]^

## 3 Progress in the MOF Applications

MOFs stand out for their unique structural designability, sustainability, and multifunctional features, such as excellent crystallinity, high porosity, huge surface area, and strong metal‐ligand interactions. This makes MOFs a very special class of materials with promising applications. In the early days, MOFs played a key role in the field of gas adsorption and storage, realizing gas separation by adjusting the pore structure. However, in recent years, the application fields of MOFs have expanded rapidly. From drug delivery to biosensing, from energy storage and conversion to energy harvesting, from catalysis to gas sensing, MOFs have shown their potential. MOFs are used as carriers for drug release, providing an innovative way for precise drug delivery. In biosensing, MOFs are used in molecular detection, protein analysis, and cell imaging, to facilitate biomedical research and diagnosis. In addition, the applications of MOFs in energy, environment, optoelectronics, and other fields are emerging. Its versatility and tunability have triggered innovations in scientific research and practical applications, demonstrating the emerging potential of MOFs in interdisciplinary research and innovation, and bringing continuous impetus to the fields of energy, environmental protection, and healthcare.

### 3.1 Gas Storage and Separation in MOFs

**Figure 3:**
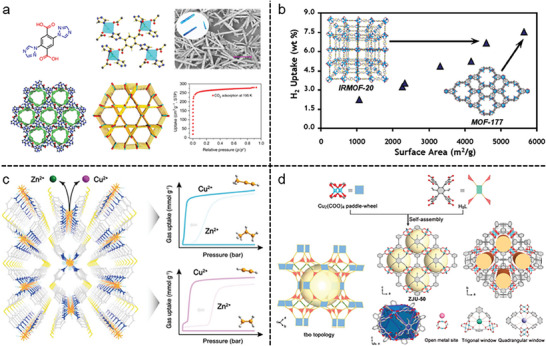
Gas storage and separation in MOFs. a) Carbon dioxide capture and conversion by an acid‐base resistant MOF. Reproduced with permission.^[^ ^101^ ^]^ Copyright 2017, Springer Nature. b) MOF for H_2_ absorption and storage. Reproduced with permission.^[^ ^105^ ^]^ Copyright 2006, American Chemical Society. c) Molecular sieving of C_2_‐C_3_ alkene from alkyne with tuned threshold pressure in robust layered MOFs. Reproduced with permission.^[^ ^118^ ^]^ Copyright 2020, John Wiley and Son. d) Engineering supramolecular binding sites in MOFs for simultaneous high C_2_H_2_ storage and separation. Reproduced with permission.^[^ ^120^ ^]^ Copyright 2022, John Wiley and Son.

There are numerous ways to properly store gas, but they all require a multistage compressor and high‐pressure tank. There is a need for a more straightforward and affordable solution because these techniques are very expensive in practice. Several substances, such as zeolite or activated porous carbons, etc., have been developed to address these problems and develop safer storage techniques. MOFs are preferred over other porous materials because of their simple preparation techniques, high surface area, numerous functionalization possibilities, and tuneable pore structure.^[^ ^100^ ^]^ For example, in the CO_2_ separation from exhaust gas produced by coal‐fired power plants, which is composed of traces of SO_x_, NO_x_, and other pollutants as well as 15–16% CO_2_, 73–77% N_2_, 5–7% H_2_O, and 3–4% O_2_, harsh environments, such as those loaded with water or moisture, are invariably present in the majority of practical sorption/separation processes (**Figure** **3**a).^[^ ^101^ ^]^ Such applications call for MOFs with respectable chemical stability. The increased surface area of MOFs with open metal sites promotes greater interaction between the metal ion and H_2_ molecule. For H_2_ storage, more than 300 MOFs have been examined. For instance, MOF‐177, which combines [Zn_4_O] clusters with 4,4',4''‐benzene‐1,3,5‐triyltribenzoate (BTB) is one of the more promising MOFs.^[^ ^102^ ^]^ It exhibits a gravimetric H_2_ uptake of 7.5 weight percent at 70 bar and 77 K because of its high surface area (5000 m^2^g^−1^) and large pore volume. However, Zn(OAc)_2_ and terephthalic acid‐based MOF‐5 (IRMOF‐1) have a BET surface area of 3800 m^2^g^−1^ and H_2_ take up 7.1 wt% at 40 bar and 77 K.^[^ ^103^ ^]^ NU‐100 has a storage capacity for excess H_2_ of 99.5 mg g^−1^ at 56 bar and 77 K, which is the highest while MOF‐210 has the greatest total H_2_ storage capacity of 176 mg g^−1^ at 80 bar and 77 K has been observed.^[^ ^104^ ^]^

Since removing trace CO_2_ from the air is important for both the environment and human health, MOFs can help lower the level of CO_2_ in the environment. For example, a custom MOF called SIFSIX‐3‐Cu was used to selectively adsorb trace CO_2_ at 74% relative humidity (Figure 3b). It was amazing that high humidity had no impact on SIFSIX‐3‐Cu's apparent selectivity at 1000 ppm CO_2_. As a result, the MOF demonstrated high stability and a potent affinity for CO_2_.^[^ ^105^ ^]^ In another study, MOF‐210 has the highest surface area (10450 m^2^g^−1^) synthesized from 4,4',4''‐[benzene‐1,3,5‐triyltris(ethyne‐2,1‐diyl)] tribenzoate (H_3_BTE), bi‐phenyl‐4,4'‐dicarboxylate (H_2_BPDC), and zinc(II)nitrate hexahydrate and showed uptake of CO_2_ 22400 mg g^−1^ (74.2 wt.%, 50 bar at 298 K). Under comparable experimental circumstances, MOF‐200 and MOF‐210 both absorb CO_2_ similarly.^[^ ^106^ ^]^

A number of other well‐known MOFs, including NU‐100 (69.8%, 40 bar at 298 K), Mg‐MOF‐74 (68.9%, 36 bar at 278 K), MOF‐5 (58 wt.%, 10 bar at 273 K), and HKUST‐1 (19.8%, 1 bar at 298 K), also exhibit significant CO_2_ absorption.^[^ ^107^, ^108^, ^109^ ^]^ Additionally, it has been demonstrated both experimentally and theoretically that the presence of polar groups like NH_2_ or free N‐containing organic heterocyclic residues on the pores aids in higher CO_2_ uptake when compared to analogies that have not been functionalized. One of the greatest instances, bio‐MOF‐11, demonstrated the effects of an N‐heterocycle on CO_2_ uptake of 15.2 wt% (at 1 bar and 298 K).^[^ ^110^ ^]^ Another MOF, Cu‐m‐TCPB derived from the organic linker 1,2,4,5‐tetrakis(3‐carboxyphenyl)benzene (*m*‐H_4_TCPB) by Yan et al. in 2014 revealed that at 77 K and 1 atm, this compound can absorb not only 24.4 cm^3^g^−1^ H_2_ and 2.3 cm^3^g^−1^ N_2_ but also 23.3 cm^3^g^−1^ acetylene, 23.1 cm^3^g^−1^ CO_2_, and 7.3 cm^3^g^−1^ at 273 K and 1 atm.^[^ ^111^ ^]^ From these demonstrations, it can be concluded that functionalized MOFs are often more effective at capturing CO_2_ than their non‐functionalized counterparts due to the introduction of specific chemical groups, known as functional groups, within the MOF structure. These functional groups can enhance the adsorption capacity, selectivity, and stability of MOFs for CO_2_ capture. First, MOF functionalization can enhance chemical affinity.^[^ ^112^ ^]^ Functional groups such as amine (‐NH_2_) or hydroxyl (‐OH) groups, can be introduced to form chemical interactions (e.g., hydrogen bonding) with CO_2_, leading to higher adsorption capacities. Apart from this, functionalization can also increase the surface area of MOFs.^[^ ^113^ ^]^ A higher surface area provides more active sites for CO_2_ adsorption, improving the overall efficiency of the material in capturing CO_2_ from a gas mixture. Besides, MOF functionalization also benefits its stability and tuning properties.^[^ ^114^ ^]^

Two isomeric anion‐pillared MOFs SIFSIX‐2‐Cu‐i and ZU‐32, with good water stability and suitable apertures (4.5–4.7), were claimed to be able to efficiently isolate propylene/propane, C_3_H_6_/C_3_H_8_ by Wang and co‐workers in 2020.^[^ ^115^ ^]^ The enhanced C_3_H_6_/C_3_H_8_ separation resulted from the ZU‐32 MOF affinity for C_3_H_6_ being higher (2.2 mmol g^−1^ for C_3_H_6_/C_3_H_8_ (50/50, v/v) mixture under ambient conditions) than those for C_3_H_8_ when compared with SIFSIX‐2‐Cu‐i and other related materials. The size and shape of MOFs via weak interactions with gases play a significant role in increasing the separation efficiency of C_3_H_6_/C_3_H_8_ mixtures.

A MOF designated by JXNU‐13‐F that was obtained by the reaction of 3,3′,5,5′ tetrakis(fluoro)biphenyl‐4,4′‐dicarboxylic (TFBPDC^2−^) and 2,4,6‐tri(4‐pyridinyl)‐1,3,5‐triazine(tpt) ligands with nickel metal center showed an excellent Xe capacity of 144 cm^3^ g^−1^ at 273 K and 1 bar due to the rapid Xe absorption and moderate Xe/Kr adsorption selectivity.^[^ ^116^ ^]^ The comparative studies with non‐fluorinated analog revealed that F groups in JXNU‐13‐F made efficient Xe uptake and high Xe/Kr separation through weak interactions as well as large polarizable characteristics and large diameter of Xe.

Lin et al. presented a good methodology for the selective adsorption of C_3_H_4_ over C_3_H_6_ through H···F hydrogen bonding interaction using a fluorous ligand in MOFs.^[^ ^117^ ^]^ The activated lanthanide MOF, JXNU‐6a that was obtained from its parent compound {[Tb_2_(TFBPDC)_3_(H_2_O)]·4.5DMF·0.5H_2_O}*_n_* (TFBPDC^2−^ = 3,3′,5,5′‐tetrafluorobiphenyl‐4,4′‐dicarboxylate ligand, JXNU‐6) had the ability to capture C_3_H_4_ with an amount of 111.2 mmol L^−1^ from the C_3_H_4_/C_3_H_6_ mixture, showing its efficient performance in gas separation study.

Flexible two‐layered fluorinated MOFs, GeFSIX‐dps‐Cu and GeFSIX‐dps‐Zn (dps = 4,4′‐dipyridylsulfide) that were two layered MOFs were created in the same year by Yang and co‐workers (Figure 3c).^[^ ^118^ ^]^ Benefiting from the expanded pore aperture and interlayer space associated with host‐guest interaction through multiple binding sites, these MOFs enabled not only an exclusion of C_3_H_6_ from C_3_H_4_ but also the exclusion of C_2_H_4_ from C_2_H_2_.

In another study, peroxo‐functionalized MOF‐74‐Fe was a reference material for ethane‐selective C_2_H_6_/C_2_H_4_ separation and showed good separation performance owing to its high C_2_H_6_/C_2_H_4_ selectivity (4.4), despite having an average C_2_H_6_ uptake capacity (74.3 cm^3^ g^−1^).^[^ ^119^ ^]^ Li et al. developed a new strategy by designing abundant supramolecular binding sites into a chemically stable HKUST‐1‐like MOF (ZJU‐50a). The MOF simultaneously achieves high C_2_H_2_ storage and selectivity, breaking the trade‐off between adsorption capacity and selectivity for C_2_H_2_/CO_2_ separation (Figure 3d).^[^ ^120^ ^]^ Several theoretical and experimental studies proved the significant role of gas storage and separation capacity of MOFs.^[^ ^120^, ^121^, ^122^, ^123^, ^124^ ^]^ The exceptional ultrahigh porosity, large surface area, tunable structure, adaptable functionalities, high thermal stability, and even exceptional chemical stability of MOFs make them a distinct and pivotal role in gas storage and separation.^[^ ^125^ ^]^ Especially, flexible MOFs undergo reversible structural transformation to increase their transport capability or selectivity of gas storage and separation.^[^ ^126^ ^]^ **Table** **1** lists some representative classes of MOFs that show gas adsorption performance.

**Table 1:** Summary on the gas adsorption of MOFs.

Reference	MOF types	Selective adsorption	surface area (m^2^g^−1^)	Adsorption capacity
[102, 127]	MOF‐177	H_2_	5000	7.5 wt% (70 bar and 77 K)
[103]	MOF‐5 (IRMOF‐1)	H_2_	3800	7.1 wt% (40 bar and 77 K)
[106]	MOF‐210	CO_2_	22 400	74.2 wt.% (50 bar and 298 K)
[107]	Mg‐MOF‐	CO_2_	∖	68.9 wt.%, 36 bar at 278 K
[108]	MOF‐5	CO_2_	∖	58 wt.%, 10 bar at 273 K
[109]	HKUST‐1	CO_2_	∖	19.8 wt.%, 1 bar at 298 K),
[110]	MOF‐11	CO_2_	∖	15.2 wt% (1 bar and 298 K)
[111]	Cu‐m‐TCPB	CO_2_	23.1	7.3 cm^3^g^−1^ at 273 K and 1 atm
[115]	SIFSIX‐2‐Cu‐i and ZU‐32	C_3_H_6_/C_3_H_8_	∖	2.2 mmol g^−1^ for separation
[116]	JXNU‐13‐F	Xe/Kr adsorption	∖	144 cm^3^ g^−1^ (Xe, 273 K and 1 bar)
[119]	MOF‐74‐Fe	C_2_H_6_	∖	74.3 cm^3^ g^−1^

From a historical point of view, rapid progress has been made in the realm of gas storage and separation owing to the tunable architecture of the framework and the controllable pore functionality. The breathing phenomena and large specific surface area render flexible MOFs promising candidates for applications in the fields of gas storage and separations. In most of the studies, MOFs serve as adsorbents or membrane materials, and therefore, future research directions need to be continued to better control the accessibility of the void space and the functionalities within the frameworks and solve several practical issues such as adsorption/desorption in tanks filled with MOF materials, the packing efficiency and increase in reusability of MOFs, etc. For the adsorption of toxic gases with MOFs, which are highly corrosive, the main target needs to be explored to afford high‐affinity binding sites within MOFs. Thus, smart MOF materials for the application of gas storage and separation can be rationally designed based on the understanding of the relationship between the porosity/structure/functionalization of MOFs and their gas storage/separation properties.

### 3.2 Catalysis in MOFs

Strong metal‐ligand interactions in MOFs can give the material persistent porosity, making it possible to totally remove solvent molecules without the structure collapsing. As heterogeneous catalysts, MOFs have demonstrated tremendous potential. The MOFs are effective catalysts because, when labile ligands are often solvent molecules and when they are removed, they leave a free coordination position on the metal.^[^ ^100^ ^]^ This is because the metal centers are not entirely blocked by organic ligands or unsaturated, i.e., labile ligands, in these MOFs. As a family of porous materials, MOFs are playing a crucial role in the development of artificial enzymes that operate in biological systems which are reviewed in detail by Simms and co‐workers.^[^ ^128^ ^]^

**Figure 4:**
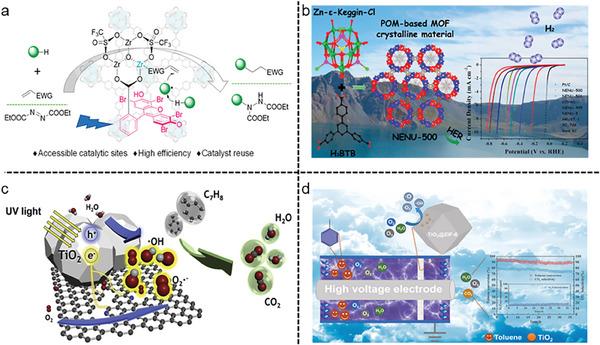
Catalysis in MOFs. a) Directed photocatalyzed hydrogen atom transfer by carbonyl‐based photocatalysts. Reproduced with permission.^[^ ^129^ ^]^ Copyright 2023, American Chemical Society. b) Polymolybdate‐based MOFs as highly active electrocatalysts for hydrogen generation from water. Reproduced with permission.^[^ ^131^ ^]^ Copyright 2015, American Chemical Society. c) MOF derivative‐TiO_2_ composite as an efficient and durable photocatalyst for the degradation of toluene. Reproduced with permission.^[^ ^132^ ^]^ Copyright 2020, Elsevier. d) Nonthermal plasma catalysis enhances the simultaneous removal of toluene and ozone over TiO_2_@ZIF‐8. Reproduced with permission.^[^ ^133^ ^]^ Copyright 2022, Elsevier.

A bifunctional photocatalyst MOF, Zr‐OTf‐EY (EY = Eosin Y dye) wherein EY acts as a Lewis acid on metal cluster nodes in MOF underwent cross‐coupling catalytic reaction of C‐H compounds with electron‐deficient alkenes or azodicarboxylate, giving rise to C‐C and C‐N products with a TON (Turn‐over number) of 1980 (**Figure** **4**a).^[^ ^129^ ^]^ The proximity between photoactive EY and nodes of MOF increased the catalytic performance ≈ 400 times. In this reaction, EY acted as a long‐lived catalyst for H atom transfer by stabilizing in the site isolation on the metal‐organic layer (MOL).

A unique bimetal complex [Zn_4_Ru_2_(bpdc)_4_.4C_2_NH_8_.9DMF]_n_ (H_2_bpdc = 4,4′‐biphenyldicarboxylic acid) created by Xu et al.^[^ ^130^ ^]^ underwent to adsorb the cobalamin derivative, heptmethyl cobyrinate perchlorate (B_12_), and the Ru(II) photosensitizer [Ru(bpy)_3_]^2+^, leading to produce the complex, B_12_‐Ru@MOF. It has been noted that hybrid MOF, B_12_–Ru@MOF can catalyze 1,2‐migration and dechlorination reactions in a solid state. This is the first instance of MOF system‐based B_12_ catalysis.

Qin et al.^[^ ^131^ ^]^ used the extremely stable polyoxometalate (POM)‐based MOFs NENU‐500 and NENU‐501 as HER (hydrogen evolution reaction) electrocatalysts in 0.5 M H_2_SO_4_ aqueous solution after rationally designing them for the reaction. Among them, NENU‐500 performed high activity for HER with a Tafel slope of 96 mV·dec^−1^ and a current density of 10 mA·cm^−2^. In addition, its HER activity was studied by DFT calculations. These two MOFs kept maintaining their performances even after 2000 cycles (Figure 4b).

Photoactive MOFs act as promising candidates in the catalytic degradation of volatile organic compounds (VOC). For instance, encapsulation of photocatalyst TiO_2_ into ZIF‐8 modified N‐doped graphite carbon GC‐N via hydrothermal synthesis produced an efficient catalyst GC‐N‐TiO_2_. It outperformed well in the photocatalytic degradation of toluene with significant durability after 20 recycling when compared to the Pt catalyst. In addition, this catalyst produced reactive active species (ROS) upon transferring photogenerated electrons to oxygen in the pyridinic N in GC‐N (Figure 4c).^[^ ^132^ ^]^

In another study, instead of using N‐doped graphite carbon, a nonthermal plasma (NTP) modified TiO_2_@ZIF‐8 catalyst was used to catalyze the photodegradation of VOCs (Figure 4d).^[^ ^133^ ^]^ Several perspectives and review articles highlighted the significant activity of MOFs toward various organic catalytic transformations.^[^ ^11^, ^12^, ^134^, ^135^ ^]^

MOFs play a distinct and significant role in catalysis due to their tunable architecture and controllable pore functionality. To increase the catalytic performance of MOFs, their pores can be decorated with catalytic sites. Furthermore, the crystalline nature of MOFs permits to analysis of the distribution of active sites within the structure and also to assess the impact of the structural framework toward catalytic activity. MOFs can be effective catalysts when the removal of labile solvent molecules leads to create a free coordination position on the active metal center. As MOFs show the potential to bridge the gap between micro‐ and mesoporous materials through a balanced mix of crystallinity, porosity, and tunability, they can demonstrate tremendous potential for commercial application in heterogeneous catalysis.^[^ ^11^, ^136^ ^]^

With the development of synthesis strategies, past decades have witnessed that MOFs remain stable in a broad range of pH and temperature and show a critical role in the conversion and selectivity of the catalytic reaction due to the inheritance of advantages (wide surface area, ultrahigh porosity, controllable structural orientation, high thermal and chemical stability, etc). Some of the articles omit the porosity which has an impact on the judgment of the actual catalytic factors. The crystalline nature of MOFs permits to examination of the distribution of active sites within the framework, and also to evaluation of the catalytic activity influenced by the framework. However, MOF‐based catalysts containing different metal active sites exhibit contradictory performance, which deserves more attention to solve systematic exploration. MOFs can become a real alternative for the removal of various pollutants when these materials are easily modulated to absorb visible light. Therefore, they can be considered as efficient photocatalysts under solar irradiation. The understanding of structural characteristics and reaction mechanism of MOF catalysts toward the catalytic activity of organic conversion under different reaction conditions will be useful in designing efficient catalysts for specific catalytic processes. MOFs have limited their practical applications in the catalysis field as they require severe reaction conditions for organic reactions owing to difficult‐to‐expose Lewis sites and incompatible pore surroundings. In addition, they have been sluggish in catching up with several reactions such as enantioselective processes, C−H activation, or olefin metathesis. During synthesis, the structure of MOFs is highly sensitive to the acidity and alkalinity of solvents, excess impurities, and reaction temperatures which will affect the morphology or crystal of MOFs. These factors impede successful applications in industry processes. Future research will focus on the creation of MOFs with improved capabilities across a range of catalytic applications.

### 3.3 Magnetism in MOFs

The term “magnetic metal‐organic frameworks” (MMOFs) refers to metal‐organic frameworks (MOFs) that exhibit magnetism when paramagnetic 3D transition metal nodes and adequate diamagnetic organic linkers are utilized.^[^ ^137^ ^]^ Usually, first‐row MOFs made of transition metals such as V, Cr, Mn, Fe, Co, Ni, and Cu have made a substantial contribution to the creation of porous molecular magnets, which permit the change in spin quantum number and magnetic anisotropy. The potential for such a wide variety of variables creates numerous obstacles in an area without boundaries.^[^ ^137^, ^138^ ^]^ The weak magnetic contact provided by close shell ligands such as oxo, cyano, and azido bridged ligands and polycarboxylic acid linkers make them suitable for this use. The framework structure of MOFs, which may have layered geometry and a shorter conjugated distance between metal clusters, is another factor in their magnetic activity. MMOFs have also been made using organic linkers, and the radicals in these linkers are what give them their magnetic properties.^[^ ^138^, ^139^ ^]^ For example, Yu and Wang reported that manganese‐based MOF, Mn_3_(THQ)_2_ (THQ = tetrahydroxyquinone) which was synthesized by using an electrostatic gating technique exhibited a high Curie temperature of 250 K in the neutral monolayer and 300 K in the hole‐doped monolayer due to the presence of a minimal‐sized ligand and square‐planar MnO coordination.^[^ ^140^ ^]^ In another study, Ni‐Glutarate based MOF [Ni_20_(H_2_O)_8_(C_5_H_6_O_4_)_20_.40H_2_O] showed ferromagnetic behavior at a curie temperature of 4K due to weak ferromagnetic interactions of Ni‐O‐Ni angle.^[^ ^141^ ^]^

**Figure 5:**
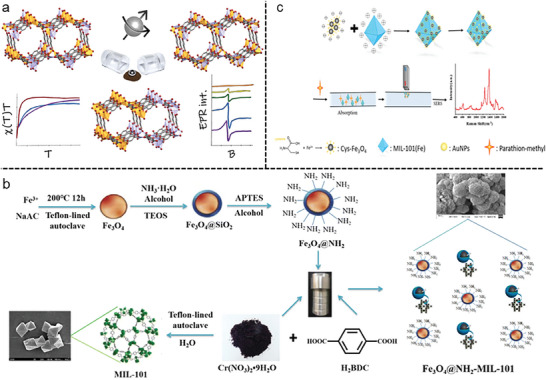
Magnetism in MOFs. a) Magnetic properties of three different binary multivariate MM‐MOF‐74 materials. Reproduced with permission.^[^ ^143^ ^]^ Copyright 2022, American Chemical Society. b) Magnetic Au/Cys‐Fe_3_O_4_/MIL‐101 is synthesized for the detection of parathion‐methyl. Reproduced with permission.^[^ ^144^ ^]^ Copyright 2021, Elsevier. c) Schematic of the preparation process of Fe_3_O_4_@NH_2_‐MIL‐101. Reproduced with permission.^[^ ^145^ ^]^ Copyright 2020, Springer Nature.

Lanthanides additionally produce magnetic metal‐organic frameworks in place of 3D transition metals. Two magnetic lanthanide organic frameworks (LnOF), with the formulas [Dy_2_(bpa)_2_(H_2_O)_3_] and [Er_4_(bpa)_4_(H_2_O)_6_]•(H_2_O), which are composed of 3D frameworks created by 3,5‐bis(4‐carboxyphenoxy) benzoate (bpa^3−^) ligands and 1D rod‐shaped metal carboxylate SBUs showed Ln^III^⋯Ln^III^ ferromagnetic interactions.^[^ ^142^ ^]^ One of the classic examples of a magnetic metal‐organic framework (MOF) family is copper(II)/zinc(II)‐MOF‐74, which is constructed via a mechano‐synthetic approach using a linker, 2,5‐dioxido‐1,4‐benzenedicarboxylic acid (H_4_dobdc) as a bridge.^[^ ^143^ ^]^ According to the result of magnetic performance, both antiferromagnetic intrachain and weaker ferromagnetic interchain play a major role in copper(II)/zinc(II)‐MOF‐74, and these results were compared with Cu‐MOF‐74 (**Figure** **5**a).

An example of Fe‐based MOF has been shown to act as the best platform in the magnetic solid phase extraction (MSPE) of pesticides in an aqueous medium.^[^ ^144^ ^]^ Fe_3_O_4_@NH_2_‐MIL‐101 MOF, which was obtained by solvothermal reaction of Fe_3_O_4_@NH_2_, H_2_BTC (BTC = benzenetricarboxylicacid) (Figure 5b) and MIL‐101 (Cr) possessed extremely high surface area (1012.7 m^2^ g^−1^), a large pore volume (0.64 cm^3^ g^−1^), and a high magnetic responsiveness (30.0 emu g^−1^). UV sensitivity studies revealed that low limits of detection (S/N = 3) of 0.13–0.86 µg L^−1^ and strong repeatability (1.8–3.1%) allowed for the achievement of a linear range that extended to 1000 µg L^−1^. A successful analysis of several pesticides in an aqueous medium using Fe_3_O_4_@NH_2_‐MIL‐101 gave the range of 70.5‐119.8% with the RSDs <8.1% and acceptable recoveries.

As a magnetic surface‐enhanced Raman scattering (SERS) substrate, Zhu and co‐workers^[^ ^145^ ^]^ methodically prepared a metal‐organic framework MIL‐101 embellished with Au nanoparticles and cysteine‐modified Fe_3_O_4_ (designated as/Cys‐Fe_3_O_4_/MIL‐101). The SERS approach successfully was used to detect Parathion‐methyl (PTM) insecticide residue in juice by making use of the magnetically induced improvement effect and Au/Cys‐Fe_3_O_4_/MIL‐101's excellent sensitivity and detection repeatability (Figure 5c). It was possible to detect PTM at a limit of detection of 5 ppb in juice with high linearity between 10^−11^ and 10^−7^ M.

A 2D MOF compound, [Gd^III^_2_(ox)_3_(H_2_O)_6_]_n_·4nH_2_O (ox = oxalate) exhibited magnetic relaxation and moderately large magnetocaloric efficiency in the supra‐Kelvin temperature region, which can be used in the slow‐relaxing magnetic materials for cryogenic magnetic refrigeration.^[^ ^146^ ^]^

The distinct role of MOFs in magnetism stems from their ability to incorporate magnetic centers either in the functional nodes or magnetic components in the pores of a crystalline MOF, leading to the formation of organized magnetic nanostructures upon keeping them well separated in space. The organized assembly of magnetic entities within MOFs provides cooperative exchange interactions between spin centers via the organic linkers, thus producing a long‐range magnetic order. Furthermore, the simultaneous implementation of strong magnetic coupling and porosity within MOF causes magnetic exchange interactions between spin centers within a short distance, while porosity benefits from the use of extended organic ligands. The use of short linkers, typically with one‐, two‐, or three‐atom bridges, metalloligands, radical organic linkers, and conjugated binding bridges tend to overlap between metal centers, leading to more efficient magnetic interactions. Especially, M–O–C–O–M or M–O–M bridges are preferable when carboxylate linkers bridge between the metal centers. By constructing metal coordination environments with high magnetic anisotropy, permanent magnets can be generated with specifically tailored structures and properties. Due to the presence of large molar magnetic entropy, rare‐earth‐based MOFs serve as potential magnetic refrigerant materials for the application of liquefaction hydrogen.^[^ ^10^, ^147^ ^]^

The combination of organic ligands with magnetic metal ions usually generates 1D–3D polymeric magnets with a high anisotropy. Especially, 2D MOFs with a kagome lattice possess strong electron‐electron interactions to generate tunable exotic magnetic phases. Because of their periodic void volume and large surface area in crystallinity, the tailorability of MOF structures might be suitable for the production of magnetic materials. Tuning of physical and chemical properties of MOFs provides a fundamental understanding of their magnetic properties upon exchanging coordination polymers and metal centers. Various experimental and theoretical studies reveal that MOFs can be magnetically investigated owing to their exotic magnetic properties such as single‐molecule magnets (SMMs) and single‐ion magnets (SIMs). The magnetic state of MOFs can be changed from ferromagnetic or antiferromagnetic to superparamagnetic upon exchanging the metal ions such as Fe, Co, and Ni in the metal cluster, which is confirmed by alternating current (AC)/direct current (DC) magnetic susceptibility and field‐dependent magnetization. There are a series of meritorious examples of magnetic MOF composites in the literature wherein several applications have been successfully employed. The combination of MOFs with magnetic nanoparticles (MNPs), i.e., magnetic framework composites (MFCs) exhibits not only numerous innovative and fascinating features but also displays a wide variety of applications in catalysis, environmental remediation, sensing, and separation, etc., because of their high selectivity, excellent dispersivity, and recyclability. Despite numerous advantages of magnetic‐MOFs, there are several critical issues, such as controlling the growth of MOF crystals on magnetic nanoparticles though these magnetic MOF composites have obvious advantages in adsorption and separation, catalysis, and biomedical applications. It should be necessary to develop efficient methods to prepare such kinds of magnetic MOF composites in a simpler and greener manner by taking sustainability into account. Owing to the magnetic exchange interactions between nearest neighbor moment carriers through exchanging metal ions or length of the ligands, these versatile and hybrid materials could offer advanced platforms for future innovative applications in the areas of magnetic switchers, sensors, micro‐electronics, device fabrication, and clinical therapies. and multifunction devices in molecular spintronics. Furthermore, the use of MOF containing switchable magnetic properties should be very informative for future information technology.

### 3.4 Light‐Emitting Diodes (LEDs) in MOFs

Light‐emitting diodes (LEDs) have a wide range of uses in lights, displays, and optoelectronic devices due to their high efficiency, extended lifespan, and low power consumption. Researchers are looking at novel materials for improving LEDs’ efficiency and color tunability due to the constant quest for better performance. The performance of LEDs can be improved by using metal‐organic frameworks (MOFs), which have distinctive qualities like tunable porosity, high surface area, and a variety of chemical functions.^[^ ^48^, ^148^ ^]^ Based on their high efficacy, energy efficiency, environmental friendliness, long persistence, and simple packaging, solid‐state LEDs have created a potential new path for effective and green illumination. which is an alternative to the enormous energy waste associated with traditional incandescent lights and the significant environmental risk associated with mercury leakage from fluorescent tubes.^[^ ^149^ ^]^ For the production of white light‐emitting diodes, there are two procedures. The first is a simple arrangement of trichromatic red, green, and blue (RGB) LEDs, and the second is a type of LED known as phosphor‐converted white light‐emitting diodes (pc‐WLEDs), which are created by coating UV LED chips with RGB phosphor or blue LED chips with yellow phosphor by chromaticity principles.^[^ ^148^, ^150^ ^]^

Lanthanide (Ln)‐doped materials are placed in a special position among luminous materials due to their abundance of *f*‐orbital configurations, low phonon energy, rich energy levels, and exceptional thermal and environmental stability. The co‐doped materials display outstanding luminescence capabilities with energy transmission processes between the lanthanide ions, resulting in dual conversion luminescence using various lanthanide dopants.^[^ ^149^, ^151^ ^]^ For example, using molar quantities of Tb^3+^ and Eu^3+^, Ln@bio‐MOF‐1 (Ln: Tb^3+^ and Eu^3+^), structural and luminescent properties and chromaticity of the compounds could be easily modified. The white emission shown by Tb/Eu@bio‐MOF‐1 (CIE coordinates: 0.328, 0.338) was extremely similar to the conventional white light (0.333, 0.333). The quantum yield of Tb/Eu@bio‐MOF‐1 was ≈ 52.9%, with a high color rendering index (CRI) of 86.2 and a low correlated color temperature (CCT) of 4725 K (Fig. 5). Based on the available results, Tb/Eu@bio‐MOF‐1 devices might be a workable luminescent material for LED applications.^[^ ^152^ ^]^

The creation of novel single‐phase warm white phosphors by MOFs is crucial for the manufacturing of LEDs. A unique class of single‐component Sm_x_Tb_y_Dy_0.2−x−y_‐based MOFs, successfully produced CIE coordinates (0.333, 0.3522) with a CRI of 86.7, a CCT of 4444 K, and good circulation of heating/cooling process. The coating of this MOF particle with commercially available LED lamps led to the formation of white light emission that can be maintained at high temperatures, indicating its potential as a single‐phase white light phosphor.^[^ ^153^ ^]^

Lanthanide single‐atom‐based CQDs‐N:Eu^3+^@MOF‐Ln^3+^ composites (CQDs = fluorescent carbon quantum dots) have been shown to have exceptional thermal stability (up to 510 °C), as well as had the capacity to release variable multicolor emissions. Importantly, by optimizing CQDs‐N:Eu^3+^@MOF‐Ln^3+^, white light emission from a single phosphor with good color quality, favorable optical performance, and tunable color temperature can be achieved. CQDs‐N:Eu^3+^ and MOF‐Ln^3+^ were used to build WLED devices, but they were also used to create warm WLED devices with a single CQDs‐N:Eu^3+^@MOF‐Ln^3+^ phosphor that had an ideal correlated color temperature (a CCT of 4035 K) and exceptional color quality (a CRI of 95).^[^ ^154^ ^]^

In another study, two Ln‐MOF phosphors, Tb(btc) and Eu(btc) were prepared in a simple wet chemical method using a mixture of H_3_btc (btc = benzene‐1,3,5‐tricarboxylate) and their respective nitrate salts to tune their luminescence for the applications in WLEDs. Upon changing the molar ratio, tuning of the emission color that retained over 90.6% of the initial value was achieved. The combination of these two MOFs with two different kinds of blue phosphors followed by packing them to create WLEDs. The CIE coordinates of these two devices were found to be (0.31, 0.33) and (0.31, 0.34), and therefore, they were both capable of emitting white light.^[^ ^155^ ^]^

**Figure 6:**
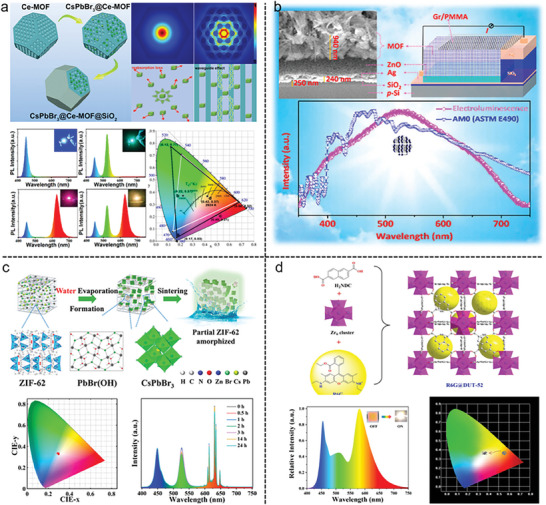
Light‐emitting diodes in MOFs. a) in situ embedding synthesis of CsPbBr_3_@CE‐MOF@SiO_2_ nanocomposites for high‐efficiency light‐emitting diodes. Reproduced with permission.^[^ ^156^ ^]^ Copyright 2022, American Chemical Society. b) Electrically driven white light emission from intrinsic MOF. Reproduced with permission.^[^ ^157^ ^]^ Copyright 2016, American Chemical Society. c) CsPbBr3 perovskite nanocrystals encapsulated in MOFs for white light‐emitting diodes. Reproduced with permission.^[^ ^159^ ^]^ Copyright 2023, American Chemical Society. d) R6G@DUT‐52 with high thermostability and luminescent were synthesized by the one‐pot hydrothermal method. Reproduced with permission.^[^ ^160^ ^]^ Copyright 2023, Elsevier.

Through the in situ growth of CsPbBr_3_PQDs (PQDs = perovskite quantum dots) in presynthesized Ce‐based MOFs followed by silane hydrolysis‐encapsulation, CsPbBr_3_@Ce‐MOF@SiO_2_ composites with improved light conversion effectiveness were prepared. Based on the modeling results, it was found that the pore boundaries of Ce‐MOFs were responsible for the production of a wave‐guiding effect, which might influence the incident PQD light, limiting it inside the bodies of Ce‐MOFs and suppressing reabsorption losses, and enhancing the total light conversion efficiency of PQDs. While this is going on, the Ce‐MOF@SiO_2_ protective shell significantly enhances stability by shielding PQDs that are implanted within from the hazardous outside environment. Additionally, the produced white light emission diode had an extremely high luminance efficiency of 87.8 lm/W (**Figure** **6**a).^[^ ^156^ ^]^

Strontium‐based MOF {[Sr(ntca)(H_2_O)_2_]·H_2_O}_n_ (naphthalenetetracarboxylic acid, H_4_ntc) has been shown to electrically driven white light emission by the combination of graphene and inorganic semiconductor (Figure 6b).^[^ ^157^ ^]^ This kind of WLED could be achieved not only by the quality nature of graphene but also by excellent band alignment of Sr‐MOF toward the semiconducting layer. This study revealed that direct WLEDs from such kind of material are rare and uncommon and will be helpful in developing solid‐state lighting devices. In the following study, authors found that Sr‐MOF exhibited continuous broadband emission at 550 nm with a CCT of 5451 K, representing a very appealing class of material to achieve near sunlight and human eye‐friendly white light.^[^ ^158^ ^]^ In order to create a compound that is more environmentally friendly than lanthanides, which are often employed in luminous materials, alkaline earth metals such as strontium can be used as metal nodes in developing WLED materials.

Utilizing a ZIF‐62 MOF as an encapsulating matrix, stable perovskite nanocrystals, CsPbX_3_NCs can be used as a light‐emitting material for the application of WLEDs. In this study, alkaline ZIF‐62 first enhanced the water‐PbBr_2_ interaction to generate PbBr(OH), which not only inactivated CsPbBr_3_NCs surface area but also restricted the perovskite from damaging. The perovskite can then be entirely sealed inside the insulating matrix by sintering ZIF‐62 in the air at a temperature of ≈ 300 °C. As a result, CsPbBr_3_/ZIF‐62 composites displayed great stability toward polar solvents, light, heat, and humidity. This nanocomposite was capable of maintaining its brightness in an aqueous medium at least for two months, thus showing its excellent color stability (Figure 6c).^[^ ^159^ ^]^

The introduction of yellow rhodamine 6G (R6G) fluorescent dyes into the DUT‐52 nanopores led to the formation of R6G@DUT‐52 composite through the hydrothermal route. With the blue emission of the DUT‐52 at 435 nm, the characteristic emission of R6G dyes at 568 nm, and the high quantum yields (QY) of 77.8%, the obtained R6G@DUT‐52 exhibited high thermostability, indicating potential application prospects as a probe for high selectivity and sensitivity of detecting Fe^3+^ ion among 12 different metals ions. To create a warm white light emitting diode (WLED) device with the CIE coordinates of (0.373, 0.364), correlated color temperature (CCT) of 4107 K, and luminous efficiency (LE) of 113.08 lm/w, R6G@DUT‐52 was combined with a commercial green phosphor powdered (GGA), which is further expressed on the 455 nm blue LED chip (Figure 6d).^[^ ^160^ ^]^

MOFs are emerging to offer a high degree of porosity which allows the inclusion of guest molecules within their structures and structural/color tunability to impart unusual luminescence behaviors. Through the selection of appropriate building blocks such as metal ions or lanthanide ions/clusters, aromatic ligands, and luminescent guests such as dye molecules, metal complexes and quantum dots, carbon dots, and perovskite quantum dots with different diameters, MOF phosphors can be flexibly designed and tuned. While the introduction of these guest molecules into MOFs, the confinement of various emitting centers into MOF via the energy transfer process along with the stability takes place, leading to efficient enhancement in the luminescent intensity of a single MOF matrix. Especially, the simultaneous incorporation of different color‐emitting lanthanide metal ions and conjugated organic ligands in the presence/absence of guest molecules facilitates the characteristic luminescent properties.^[^ ^48^, ^161^ ^]^

In contrast to organic materials used in organic light emitting displays (OLEDs), the unique properties of MOFs such as high porosity, high surface area, tunable architectures, high thermal and chemical stabilities, etc have delivered new pathways to minimize waste and energy consumption and to deliver the efficient use of local clean energy resources. The development of MOF‐based efficient emitters generated from MLCT, LMCT, LLCT, MMCT, etc., can be achieved through the availability of plenty of metal ions and organic fluorophores. Light emission can also be easily induced by the incorporation of luminescent guest molecules into MOFs via supramolecular interactions. MOFs and their composites are envisaged to provide a host media to various emissive organic dyes, providing enhancements in the lifetimes of OLED devices. However, in‐depth research on mechanisms should be devoted to providing information on MOFs and their composite design and synthesis. Several MOFs including MOF‐5, Sr‐MOF, functionalized lanthanide‐MOFs, etc., are readily available as electroluminescent MOFs to generate light emission through stimuli‐induced charge transfer. Apart from transition metal complexes, lanthanides exhibit luminescence by sensitization through covalently linked chromophores (*antenna* effect), making them valuable for OLED applications albeit they suffer from weak absorption of light. For industrialization and commercialization, it is necessary to note that the presence of defects, topology, luminescence, energy efficiency, lack of red and NIR light, stability, etc., in the MOF framework constitute important issues to take into account for further improvement of the OLEDs efficiency. The present studies give confidence that MOF‐based OLEDs exhibiting luminescence lifetimes with outstanding emission efficiencies may be developed in the solid‐state lighting industry.

### 3.5 Nanomedical Applications in MOFs

In recent years, MOFs, as an emerging and attractive organic‐inorganic hybrid material, have attracted widespread interest in the field of nanomedicine. Among them, nano‐MOFs are a special type of metal‐organic self‐assembly materials that combine the beneficial properties of bulk MOFs and nanomaterials, making them of great concern in medical applications. First, MOFs have the characteristics of tunable pore structure, controlled molecular arrangement, and easy chemical functionalization, which make them outstanding representatives of nanocarriers in the biomedical field. Secondly, MOFs exhibit rich properties from the macro to the nanoscale, providing possibilities for new applications in the nanomedical field, especially applications at the nanoscale. In addition, compared with traditional nanocarriers, MOFs have several potential advantages: 1) Their structure and composition can be adjusted according to needs, thereby constructing MOFs with different compositions, shapes, and properties. 2) Its high porosity provides sufficient space for drug loading. 3) Weak coordination bonds make it easy to degrade in biological environments. These remarkable characteristics make MOFs a promising nanomedicine platform.

MOFs were mainly used as carriers for drug delivery in early biomedical research. By immersing the prepared MOF nanocarriers in a solution containing drugs, the drugs are loaded through physical adsorption, which is usually suitable for situations where the size of the drug is smaller than the pore size of the MOF nanoparticles. Notably, the porous structure of MOFs enables them to achieve high drug loading and prolonged release. In comparison to mesoporous silica materials, MOFs have a four‐fold higher drug loading capacity, with the ability to adsorb up to 1.4 grams of ibuprofen for up to 21 days.^[^ ^100^ ^]^ Non‐toxic MOFs have become popular in biological and medical applications such as biological sensing, drug release, biological catalysis, etc., because of their flexible pore sizes, high surface area, and diverse physical and chemical properties. Excellent chemical stability in MOFs is essential in physiological conditions wherein they would be anticipated to function, such as stomach acidity, intestinal alkalinity, and the peristalsis in the esophagus, stomach, and intestines, they should have great resistance to hydrolysis/collapse.^[^ ^162^, ^163^ ^]^ The first group of trivalent metal center‐based MOFs with carboxylic acid bridging for drug delivery applications was disclosed by Férey et al.^[^ ^162^ ^]^

**Figure 7:**
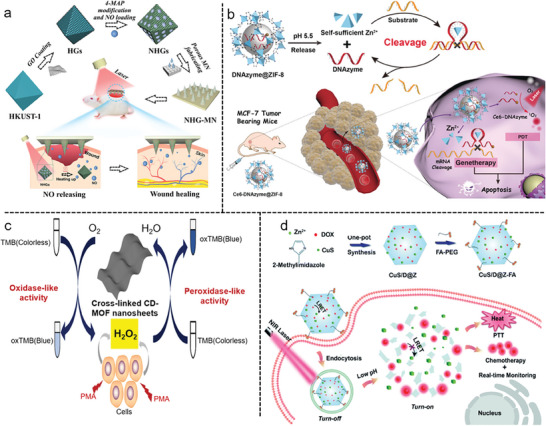
Biological and medical applications in MOFs. a) Porous MOF microneedle array patch with photothermal responsive nitric oxide delivery for wound healing. Reproduced with permission.^[^ ^164^ ^]^ Copyright 2021, John Wiley and Son. b) DNAzyme‐loaded MOFs for self‐sufficient gene therapy. Reproduced with permission.^[^ ^167^ ^]^ Copyright 2019, John Wiley and Son. c) Moisture‐resistant and green cyclodextrin MOF nanozyme based on cross‐linkage for visible detection of cellular hydrogen peroxide. Reproduced with permission.^[^ ^169^ ^]^ Copyright 2022, Springer Nature. d) Real‐time drug release monitoring from pH‐responsive CuS‐encapsulated MOFs. Reproduced with permission.^[^ ^170^ ^]^ Copyright 2022, Royal Society of Chemistry.

A photothermal‐responsive nitric oxide (NO) delivery‐enabled porous MOF microneedle patch for promoting diabetic wound healing is reported (**Figure** **7**a). The near‐infrared ray (NIR) responsive NO@HKUST‐1@GO (NHGs) microparticles were obtained upon enclosing the NO‐loadable copper‐benzene‐1,3,5‐tricarboxylate (HKUST‐1) MOF to graphene oxide (GO). While embedding NHGs in a porous poly (ethylene glycol) diacrylate microneedle patch (PEGDA‐MN), the mechanical strength of the integrated MN facilitated NO delivery into the wound site accurately and deeply. From the results obtained by the wound of type I diabetic rat model, the authors concluded that this system was able to stimulate wound healing through several processes including vascularization, tissue regeneration, and collagen deposition.^[^ ^164^ ^]^

Reynolds's group used a biomimetic catalyst, Cu‐BTTri, (BTTri = 1,3,5‐benzene‐tris‐triazole) to form nitric oxide (NO), bioactive agent from *S*‐nitrosothiols (RSNOs), an endogenous source.^[^ ^165^ ^]^ This biocatalyst exhibited perfect structural integrity in aqueous environments including phosphate‐buffered saline cell media or fresh citrated whole blood. The catalytic function was even retained upon the combination of CuBTTri MOF into biomedical grade polyurethane matrices, leading to the release kinetics in which surface flux was maintained with the corresponding therapeutic upon exposure to blood.

Bernini et al. showed how GCMC (Grand Canonical Monte Carlo) simulation can be used to predict the microscopic performance of new porous MOFs in drug delivery applications.^[^ ^166^ ^]^ They validated their simulation using the experimental data that was made available for the adsorption release of ibuprofen in MIL 53(Fe), MIL 100(Fe), and MIL 101(Cr) Cr) materials.

Metal‐organic frameworks (MOFs) have become viable drug delivery systems. A self‐sufficient MOF‐based therapeutic nanosystem based on the encapsulation of chlorin e6‐modified DNAzyme (Ce6‐DNAzyme) into ZIF‐8 was constructed for the study of gene therapy and photodynamic treatment (PDT) (Figure 7b).^[^ ^167^ ^]^ The therapeutic DNAzyme might be effectively delivered by ZIF‐8 nanoparticles (NPs) without enzymatic degradation into cancer cells. The guest DNAzyme payloads and host Zn^2+^ ions were simultaneously released due to the acidic cytoplasm of cancer cells so that they could activate gene therapy. In this study, Ce6 not only guided the effective imaging but also facilitated phototherapy through singlet oxygen.

pH‐activated DNA nanomachine allows the precise imaging of miRNAs to identify cancer cells.^[^ ^168^ ^]^ Two hairpins (Y1 and Y2) were assembled onto the surface of a ZIF‐8 MOF but under acidic circumstances, MOF disintegrated to release the adsorbed DNA hairpin molecules *in situ*, thus, creating DNA nanomachine. The target miRNA‐21 caught the released hairpins, from which catalytic hairpin assembly amplification between Y1 and Y2 took place. miRNA assay confirmed that the DNA nanomachine's limit of detection was found to be 27 pM, which is below the level of detection in living cells. The application of the DNA nanomachine in the detection of cancer cells was further supported by the scanning of miRNA‐21 in living cells.

A moisture‐resistant, peroxidase (POD), and oxidase (OXD) enzymatic active 2D cross‐linked CD‐MOF (CD = cyclodextrin) was fabricated.^[^ ^169^ ^]^ According to the POD mimic study, this biosensor was able to selectively sense H_2_O_2_ and glucose in the colorimetric study as well as H_2_O_2_ released by HepG2 cells while retaining good biocompatibility (Figure 7c). This study demonstrated its significant potential as a label‐free colorimetric probe in the early identification of cancer and the monitoring of pathological processes.

To achieve the capabilities of real‐time drug release monitoring and combined chemo‐photothermal therapy, a metal‐organic framework (MOF)‐based nanotheranostic system CuS/DOX@ZIF‐8‐FA was constructed with a combination of ZIF‐8‐FA, photothermal agents (CuS) and therapeutic drug (DOX) (Figure 7d).^[^ ^170^ ^]^ This system has been shown to improve active targeting function against cancer cells when folic acid‐conjugated polyethylene glycol (FA‐PEG) antennas were linked to MOF through coordination contacts. A theranostic agent with simultaneous tumor targeting, real‐time medication monitoring, and effective treatment was expected to improve the effectiveness of cancer therapy.

In the Outlook article, Nie and co‐workers gathered knowledge on the development of nMOFs as nano‐sensitizers for photodynamic therapy (PDT), radiotherapy (RT), radiotherapy and radio dynamic therapy (RT‐RDT), and chemodynamic therapy (CDT).^[^ ^171^ ^]^ ROS were produced under external energy stimuli or in the presence of endogenous chemical triggers when nMOFs were triggered. The abscopal effects of nMOF‐based ROS therapy enabled the extension of the local therapeutic benefits to distant tumors. Additionally, inflammatory reactions caused by nMOF‐mediated ROS production stimulated tumor microenvironments to augment cancer immunotherapy.

MOFs have several advantages to be employed in the biomedical fields: i) extremely high surface area and high porosity with commensurate high drug loading capacity, ii) easy modification of physical and chemical properties of MOFs by the judicious choice of inorganic clusters and/or organic linkers, iii) biocompatibility and high crystallinity that represents specific morphological information and definite networks to study host‐guest interactions, iv) facile diffusion of the substrates into the incorporated molecules through MOF's pores open and windows; v) biodegradability due to weak strength of coordination bonds that are critical for the controllable drug release, vi) surface engineering of MOFs significantly enhances targeted tumor therapy efficacy via stimuli‐responsive drug delivery and (5) the ability to tune the size, shape, and surfaces allows a high degree of control over drug‐binding and release kinetics.^[^ ^172^, ^173^ ^]^

Despite the potential advantages of MOFs in many aspects, research on their biological and medical applications is still in its early stages. Currently, there are many challenges that need to be overcome to achieve their widespread application in nanomedicine. First, creating nontoxic MOFs with excellent chemical stability, great biocompatibility, and suitable pore size and pore volume is a key challenge currently. Secondly, controlling the size of MOF particles is crucial. Control of particle size directly affects the interaction of MOFs with living cells, including cellular endocytosis and behavior in systemic blood circulation. In terms of drug delivery, the degradation mechanism of MOFs needs to be studied in depth. In terms of applications, further efforts to prepare MOFs that are multifunctional, specifically targeted, and capable of achieving sustained drug release are another important research direction. This will help MOFs better adapt to different treatment needs and improve treatment effects. In addition, although short‐term in vitro and in vivo studies have demonstrated the relative safety of NMOF, research on its long‐term chronic toxicity, pathways, and elimination behavior is still relatively limited. A deeper understanding of the biological responses and potential risks of NMOF in long‐term applications is crucial. Overall, multifunctional NMOF engineering is an exciting emerging field, but the above challenges still need to be overcome to better apply them in nanomedicine for disease diagnosis and treatment. For future research, breakthroughs need to be continuously made to promote the widespread application of MOFs in the medical field.^[^ ^174^ ^]^

MOFs have been widely applied in biomedical applications, specifically in drug loading and drug delivery systems, particularly for antiviral and anticancer compounds. However, solubility and long‐term stability in aqueous, alkaline, and acidic media and toxicity of the selected metal salts and organic linkers for the preparation of MOFs as well as the issues associated with biocompatibility and biodegradability are major concerns for the applications of drug delivery. Apart from this, strong interactions via pore encapsulation and surface adsorption limit their potential application. Hence, designing MOF‐drug conjugate with enhanced biostability, biocompatibility, and therapeutic efficiency is necessary to develop the smart delivery system.

### 3.6 Applications in the Energy Harvesting and Storage

In recent years, with the rapid development of the internet of things,^[^ ^175^, ^176^, ^177^ ^]^ human—machine interfaces,^[^ ^178^, ^179^ ^]^ wearable electronics^[^ ^180^ ^]^ and flexible devices,^[^ ^51^ ^]^ the demand for clean and sustainable energy has been increasing.^[^ ^181^ ^]^ The triboelectric nanogenerator (TENG), based on the coupling of triboelectrification and electrostatic induction, has emerged as one of the best solutions in the new era.^[^ ^182^, ^183^, ^184^ ^]^ The output performance is a key indicator of TENG. Various strategies have been reported recently to enhance the performance of TENG, such as selecting suitable triboelectric materials,^[^ ^185^ ^]^ charge injection,^[^ ^186^ ^]^ morphology control,^[^ ^187^ ^]^ and structural optimization.^[^ ^188^ ^]^ Among all these methods, increasing the surface charge density by selecting appropriate triboelectric materials is an effective approach to improve TENG performance.^[^ ^54^ ^]^ Currently, the triboelectric series is mainly composed of polymers and a few metals, with polymers being the main materials used in TENG fabrication.^[^ ^189^ ^]^ However, polymers face challenges in functionalization and modification. Therefore, it is necessary to explore and introduce new multifunctional materials. MOFs, due to their porous organic coordination frameworks, have been identified as highly promising triboelectric materials.^[^ ^50^, ^52^, ^54^, ^190^ ^]^ Furthermore, MOF materials possess characteristics such as large surface area, tunable porosity, flexible topology, flexible dimension, and chemical functionality, making them of wide interest in the field of energy harvesting and storage.^[^ ^2^, ^191^, ^192^, ^193^, ^194^ ^]^

**Figure 8:**
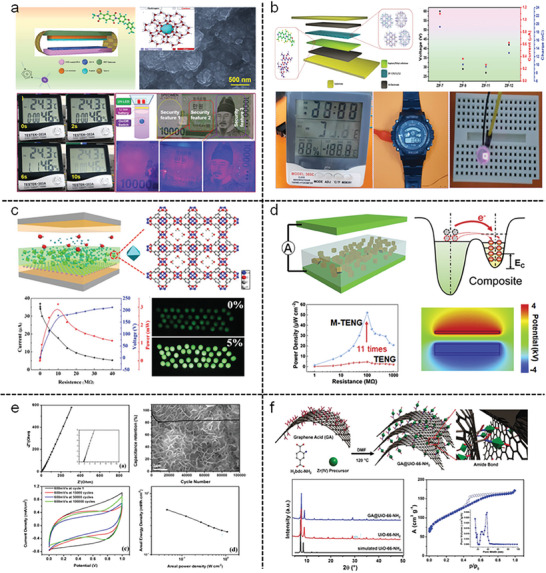
MOFs for energy harvesting and storage. a) MOF–TENG for self‐powered system and sensor applications. Reproduced with permission.^[^ ^50^ ^]^ Copyright 2019, John Wiley and Sons. b) TENG based on the ZIF subfamily materials (ZIF‐7, ZIF‐9, ZIF‐11, and ZIF‐12). Reproduced with permission.^[^ ^52^ ^]^ Copyright 2020, John Wiley and Sons. c) Humidity‐resistive TENG fabricated using MOF composite. Reproduced with permission.^[^ ^54^ ^]^ Copyright 2019, John Wiley and Sons. d) Fluorinated MOF as bifunctional filler toward highly improving output performance of TENG. Reproduced with permission.^[^ ^55^ ^]^ Copyright 2020, Elsevier. e) The MOF Ni_3_(HITP)_2_ was used as the active electrode material for supercapacitors. Reproduced with permission.^[^ ^206^ ^]^ Copyright 2019, Elsevier. f) Covalent graphene‐MOF hybrids for high‐performance asymmetric supercapacitors. Reproduced with permission.^[^ ^210^ ^]^ Copyright 2021, John Wiley and Sons.

Multiple works have reported the application of MOF in high‐performance TENG.^[^ ^53^, ^56^, ^195^, ^196^, ^197^, ^198^ ^]^ For instance, Khandelwal et al. first reported the use of zeolitic imidazole framework (ZIF) MOF‐based TENG for self‐powering systems and sensor applications (**Figure** **8**a).^[^ ^50^ ^]^ The MOF‐TENG was fabricated using zeolitic imidazole framework‐8 (ZIF‐8) and Kapton as the active materials. ZIF‐8 was grown on an indium‐doped tin oxide (ITO)‐polyethylene terephthalate (PET) substrate using a solution‐based method. Different thicknesses of ZIF‐8 films were obtained by controlling the number of cycles. The fabricated MOF‐TENG operated in the traditional vertical contact‐separation mode, with ZIF‐8 and Kapton as the positive and negative triboelectric layers, respectively. The potential of MOF as a candidate for TENG applications was confirmed through surface potential and electrical measurements. When ZIF‐8 was grown cyclically 20 times, the MOF‐TENG generated a sustainable output of 164 V and 7 µA in the vertical contact‐separation mode. The high output was attributed to the formation of high surface potential and sharp surface structures. Furthermore, Khandelwal et al. demonstrated the applicability of MOF‐TENG in driving low‐power electronic devices, counterfeit UV detection systems, and tetracycline sensors. In addition to the thickness effect, different types of ZIF subclasses also affect the output performance of MOF‐TENG. Subsequently, Khandelwal et al. reported TENG models based on ZIF subfamily materials (ZIF‐7, ZIF‐9, ZIF‐11, and ZIF‐12) (Figure 8b).^[^ ^52^ ^]^ ZIF and Kapton are used as triboelectric layers. Surface potential measurements reveal positive behavior of ZIFs toward Kapton. A series of electrical measurements found that ZIF‐7 worked best as an active material. The ZIF‐7 TENG has the highest output performance of 60 V and 1.1 µA in the vertical contact separation mode. The difference in output performance comes from different surface roughness. Finally, the group successfully drove various low‐power electronic devices such as watches, calculators, hygrometers, and UV and infrared (IR) LEDs using the capacitors charged by the ZIF‐7 TENG output. Considering the importance of non‐toxic MOFs for sustainable development, Khandelwal et al. reported a biodegradable MOF MIL88A for TENG. Electrostatic surface potential measurements indicated a relatively positive behavior of MIL‐88A. The MIL‐TENG with fluorinated ethylene propylene (FEP) as the opposite layer produced the maximum output voltage and current of 80 V and 2.2 µA, respectively. MIL‐TENG has been demonstrated for biomechanical energy harvesting and charging electronic devices such as watches.^[^ ^56^ ^]^

Mixing MOFs with polymers can also improve the output performance of TENGs.^[^ ^183^, ^199^ ^]^ Wen et al. first proposed a novel moisture‐resistant TENG technology based on HKUST‐1 and polydimethylsiloxane (PDMS) nanocomposite films (Figure 8c).^[^ ^54^ ^]^ When the weight ratio of HKUST‐1 was increased to 5%, TENG achieved the best performance. The maximum output voltage and current are 205 V and 37 µA, respectively. Furthermore, the maximum instantaneous output power (17.10 mW) can be achieved at a load resistance of 3 MΩ, which is more than 13 times higher than that of pure PDMS‐based TENG. Importantly, the performance of the composite TENG remains unchanged or becomes higher even under high humidity, while the performance of conventional TENGs drops significantly under the same conditions. This work demonstrates that MOFs are effective fillers to enhance the output performance of TENGs. Subsequently, Guo et al. introduced fluorinated MOFs (F‐MOFs) as dual‐functional fillers to develop high‐performance TENGs (Figure 8d).^[^ ^55^ ^]^ Compared with traditional fillers, F‐MOF effectively improves the amount of induced charge by introducing fluorine atoms and increasing the surface roughness. The maximum instantaneous output open circuit voltage of the prepared active material is 530 V, the short circuit current is 3.2 µA, and the power density is 52 µW cm^−2^. As a general filler, F‐MOF embedded in other polymer matrices can also improve the output performance of TENG. Hu et al. prepared a triboelectric material conforming to the nanofibrous membrane (P6‐NFM) by doping UiO‐66 into PVDF by electrospinning technology. With the weight ratio of UiO‐66 increased to 1%, the PVDF/UiO‐66 composite nanofiber‐based TENG (P6‐TENG) achieved the maximum current, voltage, and tribocharge of 4.29 µA, 52.8 V, and 22.02 nC, respectively, which are pure 6.5 times, 5.1 times and 8.0 times of PVDF‐based TENG (P‐TENG). This superior composite triboelectric material can be used as an air filter to remove airborne particulate matter (PM) by harvesting electrostatic charges from the friction between membranes.^[^ ^200^ ^]^

The huge surface area, unique ordered structure, and excellent electrical conductivity of MOF materials also make them candidates for energy storage.^[^ ^199^, ^201^ ^]^ Supercapacitors (SCs) are key components of energy storage. At present, there has been a lot of work applying MOFs in SCs.^[^ ^193^, ^201^, ^202^, ^203^, ^204^ ^]^ An example of an SC made entirely of pure MOFs as the active material was first reported by Sheberla et al.^[^ ^205^ ^]^ In this SC, Ni_3_(2,3,6,7,10,11‐hexaiminotriphenylene)_2_ (Ni_3_(HITP)_2_) is the only electrode material. The MOF‐based device exhibits an areal capacitance (18 µF cm^−2^) exceeding that of most carbon‐based materials and a capacity retention of >90% over 10 000 cycles, consistent with commercial devices. This work confirms that pure MOFs with conductive properties can be applied to future energy storage devices. Inspired by the work of Sheberla et al, the study of pure MOFs as electrode materials for SCs has attracted extensive attention. Subsequently, Nguyen et al. prepared SCs with ultrahigh cycle stability by electrophoretic deposition (EPD) (Figure 8e).^[^ ^206^ ^]^ The SC uses two‐dimensional MOF Ni_3_(HITP)_2_ as the active electrode material. The MOF‐based symmetric SC exhibits excellent electrochemical capacitive performance in the potential window of 0–1.0 V, with an areal‐specific capacitance of 15.69 mF cm^−2^. Furthermore, the Ni_3_(HITP)_2_ SC exhibited an excellent capacitance retention of 84% after 100 000 cycles. This work provides a new avenue for the development of SCs with ultra‐long life cycle stability.

Exploring MOF composites could create a high‐performance electrode.^[^ ^194^, ^207^, ^208^, ^209^ ^]^ For example, Jayaramulu et al. reported a GA@UiO‐66‐NH_2_ material covalently bonded to the basal plane of carboxylic acid‐functionalized graphene (graphene acid = GA) via an amine‐functionalized MOF (Figure 8f).^[^ ^210^ ^]^ The resulting GA@UiO‐66‐NH_2_ hybrid possesses a large specific surface area, hierarchical porosity, and interconnected conductive network. The results show that the hybrid material GA@UiO‐66‐NH_2_ acts as an effective charge storage material with a capacitance as high as 651 F g^−1^, which is significantly higher than that of conventional graphene composites. In addition, in order to achieve practical feasibility, the authors constructed an asymmetric supercapacitor with GA@UiO‐66‐NH_2_ as the positive electrode and MXene as the opposite electrode. The battery is capable of delivering a power density of up to 16 Kw kg^−1^ and an energy density of up to 73 Wh kg^−1^, almost equivalent to commercial devices such as lead‐acid and Ni‐MH batteries. At moderate load levels, the device retained 88% of its initial capacitance after 10 000 cycles. Wang et al. reported a polypyrrole (PPy)‐MOF composite fabricated from flower‐like Ni‐MOF sheets and PPy using a simple wet‐chemical approach.^[^ ^211^ ^]^ Compared with Ni‐MOF and PPy, PPy‐MOF‐modified nickel foam exhibits excellent electrochemical performance. The results show that the specific capacitance of PPy‐MOF‐modified nickel foam reaches 715.6 F g−1 at a current density of 0.3 A g−1. In addition, the authors also assembled PPy‐MOF/AC asymmetric supercapacitors. The assembled SC can deliver a high energy density of 40.1 W h kg^−1^ when the power density is 1500.6 W kg^−1^.

Compared with traditional polymer triboelectric materials, the unique properties of MOFs, such as high surface area, porosity, and tunable pore size, offer entirely new possibilities for developing high‐performance TENGs. By designing and modifying MOF materials, key parameters such as surface charge density, dielectric constant, and charge distribution can be further improved. In addition, the preparation of MOF/polymer composite materials can increase the surface roughness and surface potential of the composite film. These characteristics make MOF a highly potential triboelectric material. Although MOF‐based TENGs have been developed, research on MOF‐TENGs is still in its infancy and requires further in‐depth research. Some of the key issues require further research, including revealing the working mechanism of MOF‐TENG, the correlation between different MOF designs and TENG output performance, and realizing batch preparation of MOF‐TENG. In addition, combining MOFs with TENG for self‐powered gas adsorption and sensing, wearable sweat monitoring, and supercapacitors are also important research directions. These efforts are expected to usher in a new era of TENG equipment, providing innovative solutions for energy harvesting and self‐power technology.

### 3.7 Applications in the Gas Sensing Layer on MEMS Devices

Micro‐Electro‐Mechanical Systems (MEMS)^[^ ^212^, ^213^ ^]^ are a vital technology that has permeated many facets of daily life. They are miniaturized devices integrating electrical and mechanical elements, fabricated using integrated circuit (IC) processing techniques. Their small scale allows them to offer significant advantages over their macroscopic counterparts, including lower cost, lower power consumption, higher performance, and greater integration density. MEMS finds applications in a broad range of areas, such as medical technology,^[^ ^214^, ^215^, ^216^ ^]^ telecommunications,^[^ ^217^ ^]^ consumer electronics,^[^ ^218^ ^]^ and automotive systems.^[^ ^219^ ^]^ Innovations like inertial measurement units^[^ ^220^ ^]^ in smartphones, pressure sensors^[^ ^221^ ^]^ in cars, micro‐mirrors^[^ ^222^ ^]^ in projection systems, and MUTs^[^ ^223^, ^224^, ^225^, ^226^, ^227^, ^228^, ^229^, ^230^, ^231^, ^232^ ^]^ as fingerprint sensors/medical imaging systems all demonstrate the transformative power of MEMS technology.

**Figure 9:**
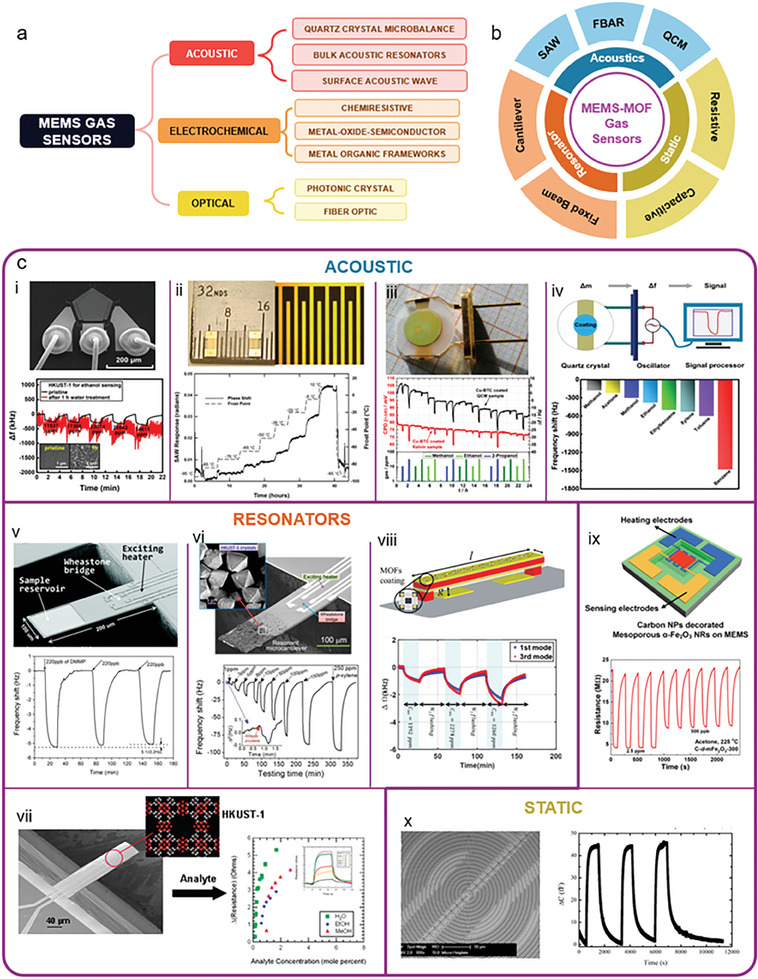
Gas sensing using Microelectromechanical Systems (MEMS) integrated with MOF. a) Common MEMS‐based gas sensing mechanisms. b) Pictorial segmented depiction of available MEMS‐MOF‐based gas sensing technologies. c) MEMS‐MOF gas sensors. i) FBAR‐based gas sensor. Reproduced with permission.^[^ ^234^ ^]^ Copyright 2020, American Chemical Society. ii) SAW‐based gas sensor. Reproduced with permission.^[^ ^235^ ^]^ Copyright 2012, American Chemical Society. iii,iv) QCM‐based gas sensor. Reproduced with permission.^[^ ^236^ ^]^ Copyright 2014, American Chemical Society. Reproduced with permission.^[^ ^237^ ^]^ Copyright 2019, Elsevier. v–vii) Cantilever‐based gas sensor. Reproduced with permission.^[^ ^238^ ^]^ Copyright 2019, Royal Society of Chemistry. Reproduced with permission.^[^ ^239^ ^]^ Copyright 2016, American Chemical Society. Reproduced with permission.^[^ ^240^ ^]^ Copyright 2008, American Chemical Society. viii) Fixed beam‐based gas sensor. Reproduced with permission.^[^ ^241^ ^]^ Copyright 2018, Elsevier. ix) Hybrid electrode‐based gas sensor. Reproduced with permission.^[^ ^242^ ^]^ Copyright 2022, Elsevier. x) IDE‐based capacitive gas sensor. Reproduced with permission.^[^ ^243^ ^]^ Copyright 2019, MDPI.

MEMS gas sensors embody a major application of MEMS technology, effectively playing a crucial role in detecting and identifying a wide range of gases.^[^ ^233^ ^]^ These miniaturized devices are specially designed to detect the presence and concentration of different gases in the environment, which is of paramount importance in several scenarios, ranging from environmental monitoring to personal safety. Characterized by their remarkably small size, low power consumption, and high sensitivity, MEMS gas sensors offer several advantages that make them ideal for use in a variety of settings. For instance, they are used extensively in industrial settings to monitor and control the emission of hazardous gases. They are also a critical component in indoor and outdoor air quality monitoring systems, where they detect and measure pollutant gases to ensure healthy living and working conditions. Moreover, in the realm of IoT, they are being increasingly integrated into smart home devices for automatic control of air quality. The core component of a MEMS gas sensor is typically a micro‐hotplate, which is coated with a chemically sensitive layer. This layer changes its electrical properties upon exposure to specific gases. For instance, it can detect harmful gases such as carbon monoxide (CO), nitrogen oxides (NOx), or volatile organic compounds (VOCs), thus providing an essential tool for maintaining environmental safety and health protection. Moreover, their potential for mass production using established semiconductor processes allows for widespread, cost‐effective deployment. **Figure** **9**a depicts the types of various gas sensors.

There has been a recent trend in the development of MEMS‐coated MOF sensors for gas‐sensing applications. On a careful literature survey, it was found that the MEMS‐MOF gas sensors can be divided into three broad categories depending on the underlying mechanism of gas sensing. The three divisions are bulk wave acoustics, structural resonant vibration, and electrostatics as depicted in Figure 9b. The domain of bulk acoustics‐based MEMS‐MOF gas sensors can be divided into further three sub‐branches – a) thin‐film bulk acoustic resonators (FBAR), b) surface acoustic wave (SAW) devices, and c) Quartz crystal microbalance (QCM). The distinct role of MOFs involves their ability to selectively adsorb gas molecules, leading to measurable changes in physical or chemical properties that can be detected and quantified. Specifically, MOFs have a highly porous structure with a large surface area, allowing them to adsorb gas molecules onto their surfaces. This adsorption process is selective based on the specific interactions between the MOF and the target gas molecules. A scanning electron micrograph (SEM) for a FBAR is depicted in Figure 9ci which was used in ethanol sensing applications. A limit of detection of 1 ppm was obtained by using HKUST‐1 MOF deposited on the surface of the FBAR. The device used the shift in the bulk resonant frequency as the sensing to gas concentration parameter.^[^ ^234^ ^]^ The next device is the SAW which is depicted in Figure 9cii which was used for water vapor sensing applications. It consists of IDT made on quartz substrate and has a detectability of 20 ppm of water vapor when coated with HKUST‐1. The device used the shift in the transmitted wave's phase as the sensing to gas concentration parameter.^[^ ^235^ ^]^ The next acoustic device is the QCM as depicted in Figure 9ciii which used HKUST‐1 coating to detect methanol, ethanol, 1‐propanol, and 2‐propanol with a sensitivity of 0.05, 0.2, 0.17, and 0.13 Hz/ppm respectively. The mechanism used to perform the gas sensing was the change in frequency with a limit of detection obtained to be 2–50 ppm.^[^ ^236^ ^]^ A similar device using QCM was also made (see Figure 9civ) which uses four different MOFs such as MOF‐14, HKUST‐1, MOF‐177, and MOF‐74 on the surface of the sensor to sense Benzene concentration in the atmosphere. An outstanding limit of detection of 150 ppb was observed, with a detectable frequency change of 1200 Hz at 80 ppm.^[^ ^237^ ^]^ The next category of MEMS‐MOF gas sensors are the resonators in the form of cantilevers and fixed micro‐beams. In the domain of micro‐cantilevers, an outstanding sensor capability was demonstrated by fabricating a silicon‐based cantilever (Figure 9cv) having a provision for a sample reservoir, into which UiO‐66 MOF was directly pattern deposited. A limit of detection of 5 ppb for detecting organophosphorus compounds was demonstrated using a Wheatstone bridge‐assisted frequency shift measurer.^[^ ^238^ ^]^ Another work on cantilevers as shown in Figure 9cvi has demonstrated the detection of Xylene molecules by employing HKUST‐1 with a limit of detection of 500 ppb. Frequency shift was the parameter of detection when the gas concentration was changed.^[^ ^239^ ^]^ A third work on cantilevers as shown in Figure 9cvii depicts the use of HKUST‐1 along with a resistive sensing mechanism to sense the presence of water vapor, ethanol, and methanol respectively.^[^ ^240^ ^]^ A fourth toward this direction was with the creation of a fixed beam‐based frequency shift device which was coated with 2D periodic porous Cu(bdc).xH_2_O to detect the presence of water vapor in the atmosphere as shown in Figure 9cviii. The sensor demonstrated a sensitivity of 0.65 Hz/ppm having a limit of detection of 3 ppm.^[^ ^241^ ^]^ In the domain of electrostatics, the first work was toward the development of a hybrid electrode pattern (see Figure 9cix) which was coated with carbon nanoparticle decorated mesoporous αFe_2_O_3_ which could detect acetone in the atmosphere to a limit of 500 ppb by using the resistive mechanism.^[^ ^242^ ^]^ The second work was toward the development of an interdigitated electrode‐based capacitive sensor (see Figure 9cx), which when coated with ZIF‐8 could detect multiple organic vapors of methanol, ethanol, and water with a detectability of 100 ppm.^[^ ^243^ ^]^

### 3.8 Applications in the Gas Trapping Layer for Mid‐IR Gas Sensing

MOFs hold great promise for gas sensing due to their unique properties and tunability. First, MOFs possess an exceptionally high surface area and porosity, providing ample sites for gas molecules to adsorb.^[^ ^244^, ^245^, ^246^, ^247^ ^]^ This characteristic enables efficient gas capture and allows for high sensitivity in gas sensing applications.^[^ ^248^, ^249^ ^]^ The large surface area also enhances the interaction between the MOF and the target gas molecules, leading to improved detection capabilities. Then, MOFs offer the advantage of a tunable pore structure. By selecting appropriate metal nodes and organic linkers, researchers can design MOFs with specific pore sizes, shapes, and functionalities.^[^ ^250^ ^]^ This tunability allows for the customization of MOFs to selectively adsorb target gases while excluding or minimizing the interaction with interfering gases.^[^ ^251^ ^]^ The ability to tailor MOFs for specific gas molecules enhances the selectivity and reliability of gas sensing. Besides, MOFs exhibit selective adsorption properties, allowing them to differentiate between different gas molecules. The coordination sites within MOFs can interact selectively with specific gas molecules based on their size, shape, polarity, or chemical properties. Despite these advantages, signal transduction is required to detect changes within MOFs, making sensing applications of MOFs challenging and involving multidisciplinary techniques.^[^ ^252^ ^]^

Mid‐infrared nanoantennas are nanostructures specifically designed to interact with electromagnetic radiation in the infrared (IR) region of the electromagnetic spectrum.^[^ ^253^, ^254^, ^255^, ^256^, ^257^, ^258^ ^]^ They are tailored to efficiently manipulate and control light at subwavelength scales, enabling enhanced light‐matter interactions in the IR regime.^[^ ^259^, ^260^, ^261^, ^262^ ^]^ This enhanced light‐matter interaction facilitates more efficient energy transfer, enabling sensitive detection of gas molecules adsorbed within MOFs. By placing MOF structures in the vicinity of nanoantennas, the localized electromagnetic fields can interact with the gas molecules, leading to measurable changes in the optical response of the system. Increased absorption and stronger vibrational signals can be obtained when using nanoantennas as the signal transduction of MOFs. This effect is also called surface‐enhanced infrared absorption (SEIRA).^[^ ^263^, ^264^, ^265^ ^]^ Nanoantennas offer flexibility in their design and resonance properties, enabling tuning to specific wavelengths or frequencies of interest.^[^ ^265^, ^266^ ^]^ This tunability allows for the selective excitation and detection of gas molecules with specific absorption or vibrational spectra within the MOF. Multiple nanoantennas with different resonant frequencies can be integrated into a single sensor, enabling multiplexed gas sensing and the simultaneous detection of multiple gases.

**Figure 10:**
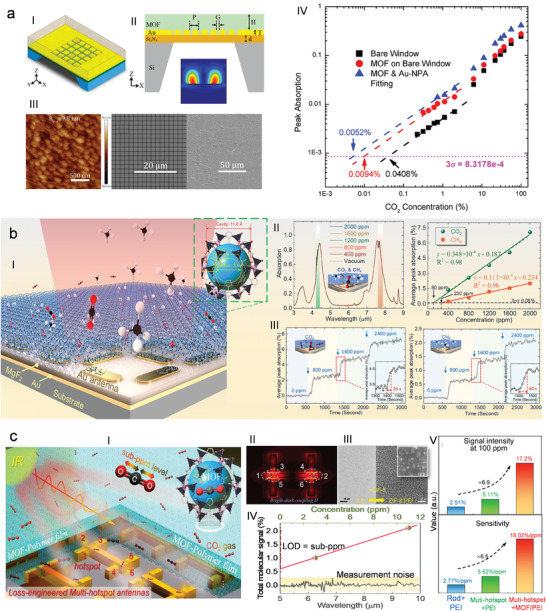
Mid‐infrared gas sensing applications in MOFs. a) CO_2_ gas detection using MOF‐nanoantenna‐integrated sensing platform. Reproduced with permission.^[^ ^267^ ^]^ Copyright 2018, American Chemical Society. i): Top view of the device. ii: Cross‐sectional view. iii): AFM image and SEM image of the device before and after coating the MOF thin film. iv): Detection limit of the platform. b) Multi‐gas detection using MOF‐nanoantenna platform. Reproduced with permission.^[^ ^26^ ^]^ Copyright 2020, John Wiley and Sons. i): Schematic representation of the multi‐gas sensing platform. ii: Steady sensing characteristics. iii): Dynamic sensing characteristics. c) Ultra‐sensitive gas detection using MOF/PEI‐nanoantenna‐integrated sensing platform. Reproduced with permission.^[^ ^25^ ^]^ Copyright 2022, Springer Nature i): Schematic view. ii): Multi‐hotspot strategy of the nanoantenna design. iii): SEM images showing the nanoantennas with PEI film (left panel) and ZIF‐8‐PEI hybrid film (right panel). iv): Steady sensing characteristics. v): Performance enhancement.

Chong *et al.* conducted an experiment involving a suspended Si_3_N_4_ nanomembrane device that integrated plasmonic nanoparticle antennas with a zeolitic imidazolate framework (ZIF‐8) film (**Figure** **10**a‐i, iii).^[^ ^267^ ^]^ This device demonstrated the capability to simultaneously amplify the localized optical field and concentrate CO_2_ from the surrounding environment. In contrast to conventional SEIRA sensing, which relies on localized hot‐spots generated by plasmonic nanostructures, the device operated near the coupled‐mode region between surface plasmon polaritons at the interfaces of the bottom metal/Si_3_N_4_ and top metal/MOF layers (Figure 10a‐ii). This arrangement enabled a significant enhancement of the optical field throughout the entire MOF film. Through the combined effects of MOF gas concentration and plasmonic field enhancement, on‐chip gas sensing of CO_2_ was successfully demonstrated, yielding enhancement factors exceeding 1800 times within a 2.7 µm thin film. Furthermore, the estimated detection limit for CO_2_ was ≈52 ppm (Figure 10a‐iv).

Through the multi‐resonant design of the nanoantenna, the MOF‐based sensor can realize multiple gas detection. For instance, Zhou *et al.* have successfully created an efficient gas sensor that combines a porous MOF with a metamaterial absorber‐based SEIRA platform (Figure 10b‐i).^[^ ^26^ ^]^ This integration enables the sensor to detect greenhouse gases in a rapid and comprehensive manner. The SEIRA platform offers substantial local near‐field intensity enhancements, exceeding 1500‐fold for both sensing bands. The MOF developed in this study exhibits remarkable selectivity and reversibility in sorbing and desorbing both CO_2_ and CH_4_. By leveraging the near‐field enhancement capabilities of the SEIRA technique and the selective trapping properties of the MOF, the MOF‐SEIRA platform enables simultaneous on‐chip sensing of CO_2_ and CH_4_. For the sensing behavior at steady state, the sensitivity of the platform is 0.0348‰ ppm^−1^ (CO_2_) and 0.0113‰ ppm^−1^ (CH_4_), respectively (Figure 10b‐ii). For the dynamic sensing characteristics, the response time is estimated ≈35 s for CO_2_ and ≈ 60 s for CH_4_ (Figure 10b‐iii).

Since the MOF adsorption to gas is a kind of physical adsorption, when it comes to ultra‐low concentration gas detection, the performance of MOF sensors is limited. Combining chemisorption and physisorption is a promising solution. Zhou *et al.* propose a novel approach that integrates hybrid MOF‐polymer physi‐chemisorption mechanisms with IR nanoantennas to achieve highly selective and ultrasensitive CO_2_ detection (Figure 10c‐i).^[^ ^25^ ^]^ To enhance the adsorption capacity for trace amounts of gas molecules, amino groups are incorporated into the MOF structure to introduce chemisorption, while still maintaining the structural integrity necessary for physisorption (Figure 10c‐iii). Additionally, a multi‐hotspot nanoantenna strategy is suggested to increase the number of hotspots and to keep high intensity near the field (Figure 10c‐ii). As a result, the modified MOF‐polymer hybrids exhibit enhanced capability in capturing CO_2_ gas compared to pure MOFs, with a sensitivity of 0.18%/ppm, which is 45 times higher than that of pure MOFs. Moreover, through the co‐design and optimization of the nanogap position and the dark mode antenna dimensions, the multi‐hotspot nanoantennas exhibit a five‐fold higher enhancement of molecular signals compared to conventional nanorod antennas. These advancements in MOF modification and nanoantenna optimization provide a competitive advantage to our strategy in state‐of‐the‐art CO2 gas sensors. This includes achieving a low limit of detection (sub‐ppm) (Figure 10c‐iv), high sensitivity (18%/ppm), high intensity (17.2%) (Figure 10c‐v), high selectivity, and enabling nm‐level optical interaction lengths for the purpose of miniaturization.

### 3.9 Applications in the Detection of Biomolecules

**Figure 11:**
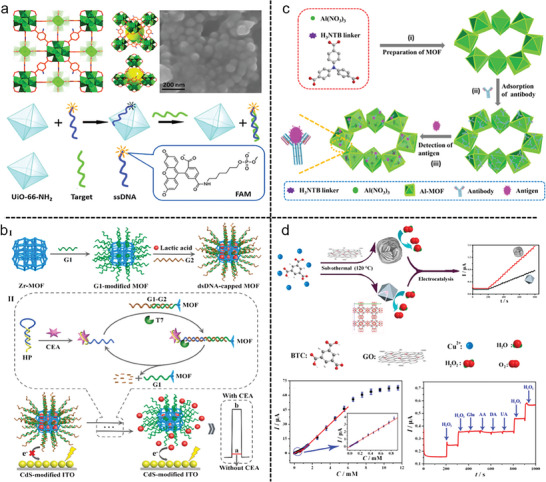
MOFs for biomolecule detection. a) Amine‐functionalized MOFs for DNA detection. Reproduced with permission.^[^ ^277^ ^]^ Copyright 2014, Royal Society of Chemistry. b) Nucleic acid‐functionalized MOFs for photoelectrochemical biosensing. Reproduced with permission.^[^ ^278^ ^]^ Copyright 2021, Elsevier. c) Highly stable Al‐based MOFs as biosensing platforms. Reproduced with permission.^[^ ^280^ ^]^ Copyright 2017, Elsevier. d) GO@HKUST‐1 for enzyme‐free detection of hydrogen peroxide in biological samples. Reproduced with permission.^[^ ^282^ ^]^ Copyright 2016, American Chemical Society.

In addition, MOFs have also played a huge role in the development of biosensor platforms, which are expected to pave the way for accurate and sensitive detection of various analytes. In biosensing applications, modified MOFs can be used as substrates for covalent or physical attachment of specific materials as bioreceptors. MOFs‐based acceptor substrates can be used to improve optical, chemical, or electrical signals. Importantly, the modified MOFs are able to recognize specific molecules such as nucleic acids, proteins, or cells with higher precision. In addition, low toxicity, high water stability, good biodegradability, and biocompatibility are also considered to be the remarkable properties of MOFs in the field of biosensing.^[^ ^268^ ^]^ Currently, there have been several reports on functionalized MOFs for biosensing.^[^ ^7^, ^269^, ^270^, ^271^, ^272^, ^273^, ^274^, ^275^, ^276^ ^]^ For example, Zhang et al. developed amine‐functionalized MOFs to realize a fluorescent sensing platform with highly selective detection of DNA (**Figure** **11**a).^[^ ^277^ ^]^ This sensing system can distinguish complementary and mismatched DNA sequences, down to single base mismatches, with high selectivity and good reproducibility. In contrast, UiO‐66, which has a similar structure without amino groups, cannot achieve such a function. Given the high stability and simple and scalable synthesis using inexpensive reactants, UiO‐66‐NH_2_‐based assays hold great promise for practical applications in the analysis of clinical samples. Liu et al. developed a novel nucleic acid‐functionalized MOF photoelectrochemical biosensor for ultrasensitive and selective determination of carcinoembryonic antigen (Figure 11b).^[^ ^278^ ^]^ In this biosensor, MOFs are used as carriers for loading electron donors, while double‐stranded DNA (dsDNA) is used as the molecular gate of the pores. These dsDNA‐capped MOFs exhibit specific responses to CEA. Utilizing the high loading capacity of MOFs and the excellent amplification efficiency of the T7 exonuclease‐assisted recovery process, the proposed biosensor enables ultrasensitive CEA detection. The detection limit of the biosensor for CEA is 0.36 fg mL^−1^, and the linear range is 1.0 fg mL^−1^ 10 ng mL^−1^. Therefore, MOFs‐based PEC biosensors provide a new detection scheme for ultrasensitive detection of biomarkers, which is of great significance in early clinical disease diagnosis and biosensing.

MOFs can be coupled with some transition metal ions, such as Zn^2+^, Fe^3+^, Cr^3+,^ and Cu^2+^. These metals endow MOFs with flexible coordination and different geometries. The various structures and shapes of the pores enhance the selectivity for the size‐dependent detection of different analytes. Therefore, changing the coupled metal ions in MOFs can change the selectivity and sensitivity of MOFs. Recently, Dang et al. proposed an enzyme‐free sensor integrating AuNPs and MOFs for highly sensitive and selective H_2_O_2_ detection.^[^ ^279^ ^]^ In this work, Cu‐based MOF (Cu‐MOF) was synthesized under ionization heat conditions and further grafted ammoniated gold nanoparticles (AuNPs‐NH_2_) to prepare AuNPs‐NH_2_/Cu‐MOF composites. Using this composite‐modified glassy carbon electrode (GCE), a highly sensitive and selective electrochemical enzyme‐free sensor for H_2_O_2_ detection was realized. The LOD of the sensor for H_2_O_2_ is as low as 1.2 µM, and the sensitivity is as high as 1.71 µA cm^−2^ µM. The AuNPs‐NH_2_/Cu‐MOF/GCE sensor can effectively detect H_2_O_2_ in human cervical cancer cells by adding ascorbic acid as a stimulant. Liu et al. proposed Al‐MOF‐based electrochemical immunosensors using 4,4',4''‐nitrilotribenzoic acid (H_3_NTB) as an organic linker (Figure 11c).^[^ ^280^ ^]^ The fabricated sensors exhibit excellent thermal and physicochemical stability, high electrochemical activity, and good biocompatibility. Based on the above properties, the sensor can be used to detect vomitoxin and salbutamol at concentrations as low as 0.70 and 0.40 pg mL^−1^. This new strategy has great potential in the detection of toxic and harmful residues in food.

Although MOFs have important advantages, they also have some limitations, such as poor electrical conductivity and structural instability in aqueous solutions. Combining MOFs with other materials can effectively overcome the shortcomings of pure MOFs.^[^ ^268^ ^]^ For example, Wang et al. successfully prepared carbon‐functionalized MOFs (C/Al‐MIL‐53‐(OH)_2_) using solvothermal technology.^[^ ^281^ ^]^ The composite was coated with a Nafion film to form a Nafion/C/Al‐MIL‐53‐(OH)_2_ modified glassy carbon electrode. The modified electrode was then used as a novel electrocatalyst for the oxidation of dopamine (DA). Under the synergistic effect of various materials, the voltammetric response of the modified electrode to DA showed a significant enhancement. The results show that the response peak current of the electrode has a linear relationship with the DA concentration in the range of 3.0 × 10^−8^ to 1.0 × 10^−5^ mol L^−1^. The detection limit and quantification limit of DA is 0.8 × 10^−8^ mol L^−1^ and 2.6 × 10^−8^ mol L^−1^, respectively. Lin's group fabricated a unique hierarchical flower‐shaped graphene oxide (GO)@HKUST‐1(Hong Kong University of Science and Technology‐1) nanocomposite by solvothermal method for H_2_O_2_ sensing in biological fluids (Figure 11d).^[^ ^282^ ^]^ Morphological and structural characterization revealed that GO, as an effective structure‐directing agent, could induce HKUST‐1 to transform from an octahedral structure to a hierarchical flower shape. Compared with pristine HKUST‐1, the synthesized flower‐shaped GO@HKUST‐1 not only has a larger pore volume and pore size but also exhibits stronger redox activity. Electrochemical analysis indicated that the GO@HKUST‐1 nanocomposite exhibited excellent performance for non‐enzymatic H_2_O_2_ detection with a fast response (<4 s), a wide linear range (1.0 µM to 5.6 mM), and a LOD = 0.49 µM.

Different from the above methods, using MOFs as substrates for enzyme immobilization is considered to be a promising strategy. Compared with other methods, MOFs combined with enzymes have many advantages, such as larger surface area, higher porosity, and better stability.^[^ ^283^ ^]^ Enzyme immobilization in MOFs is achieved through bond formation with the enzyme or its interaction with the material.^[^ ^284^, ^285^ ^]^ There are various methods to immobilize enzymes on MOFs, including surface attachment, pore trapping, covalent attachment, and co‐precipitation.^[^ ^286^ ^]^ Surface attachment is the adsorption of enzymes onto the material surface through bonding (electrostatic interactions, van der Waals forces, and hydrogen bonds).^[^ ^287^, ^288^, ^289^ ^]^ The main advantages of this method are its ease of implementation and low cost. Since the immobilization process uses physical adsorption, the conformation of the enzyme does not change. However, enzymes immobilized on MOFs via physical adsorption often exhibit low operational stability. Another approach is to trap enzymes inside the pores of MOFs.^[^ ^290^, ^291^, ^292^, ^293^ ^]^ Typically, enzyme entrapment in the mesopores of MOFs increases the stability of enzymes under adverse environmental conditions. A covalent bond is considered to be one of the strongest chemical interactions between an enzyme and a support.^[^ ^294^, ^295^, ^296^, ^297^ ^]^ The presence of amino groups on the enzyme structure allows it to be covalently linked to MOFs, thereby preventing enzyme leaching from MOFs. However, this method reduces its flexibility. This can harden the enzyme, or even denature it. Co‐precipitation is a recent approach aimed at encapsulating enzymes within MOF structures. The main advantage of this process is that higher quantities of enzymes can be added to MOFs.^[^ ^298^ ^]^

Generally, MOFs have porosity, high specific surface area, adsorption capacity, high catalytic ability, and excellent loading capacity. These organic‐inorganic hybrid materials have recently attracted widespread attention in the field of biosensing. MOFs as host matrices have been successfully used to stabilize molecules in unnatural media while maintaining their activity and functional groups unchanged. MOFs with diverse functional groups provide opportunities for chemical interactions with different biomolecules and can encapsulate analytes or enzymes by modulating porosity and pore size distribution, thereby improving their biological activity. However, although MOFs show great potential in the field of biosensing, their synthesis and performance still face some challenges and problems. To promote the rapid development of MOFs in biosensing applications, the following are some key issues and improvement directions: 1. Synthesis method improvement. More efficient synthesis methods need to be researched and developed to prepare small‐sized MOFs on a large scale. 2. Conductivity improvement. In some biosensing applications, MOFs need to show a certain degree of conductivity to achieve electronic conduction. 3. Selectivity and specificity. To achieve high selectivity in biosensing, MOFs need to be designed and synthesized with specific functional groups that can interact with target molecules and recognize their presence. 4. Cost reduction. To promote the widespread use of MOFs in biosensing applications, it is necessary to reduce their preparation costs so that they can be prepared on a large scale and become more competitive in practical applications. In summary, MOFs have broad application prospects in the field of biosensing, but further research and improvements are needed to overcome existing challenges and promote their rapid development in future biosensor platforms.

## 4 Future Trends

Metal‐organic frameworks (MOFs) have attracted a lot of interest lately because of their distinctive characteristics and prospective uses. Several potential trends and horizons hold promise for further research and investigation as our knowledge of MOFs continues to grow. Some of them consist of improved stability and structure understanding even though MOF research has advanced, there are still unanswered questions about their stability and structure. Future research will concentrate on figuring out what causes the crystal structure to degrade over time and coming up with tactics to make it more stable. This will make it possible to design and synthesize MOFs with increased durability and long‐term stability. Future research will focus on customizing the features of MOFs for particular applications because of their incredible adaptability. Researchers can tailor MOFs to exhibit desired qualities by choosing suitable metal ions, organic linkers, and functional groups. For instance, in the field of gas storage and sensing, adjusting the pore size and volume of MOFs can achieve selective adsorption of gases of different sizes, thereby enhancing the efficiency and selectivity of gas storage. This holds significant implications for applications in energy storage and environmental monitoring. In the catalysis domain, customizing the structure of MOFs can effectively improve the efficiency and selectivity of catalytic reactions. Researchers can tailor MOFs based on the requirements of specific catalytic reactions, choosing suitable metal ions and organic ligands. By adjusting parameters such as pore size and surface functional groups, they can optimize the catalyst's performance, thereby advancing green and efficient sustainable chemical synthesis. In the fields of nanomedicine and biosensing, researchers can manipulate the structure of MOFs by carefully selecting metal ions, organic linkers, and functional groups. This customization enables precise drug delivery, targeted therapy, and highly selective detection of biomolecules. Such tailored approaches not only enhance the precision of treatments but also lay the groundwork for personalized and precision medicine. Overall, tailored MOFs structures will play a key role in future research and applications, providing more efficient, precise, and controllable material solutions for various fields, and promoting the continuous progress of science and technology. In addition, the interdisciplinary expansion of MOF research has encouraged complementary skills from chemistry, materials science, physics, and engineering broadening the scope of MOF applications. This will open the door for the creation of MOFs with improved capabilities across a range of applications.

MOFs have demonstrated potential in energy storage and conversion applications, electrical devices, including batteries, supercapacitors, and catalysis, etc. In order to maximize their effectiveness in various applications, MOFs with high surface areas, specialized pore sizes, and suitable redox characteristics will be developed in the future. Technologies for storing and converting renewable energy might advance as a result. MOFs make excellent adsorption and separation candidates for environmental and industrial applications because of their remarkable adsorption and separation characteristics. Future studies will concentrate on utilizing these characteristics to tackle environmental issues such as carbon capture, gas separation, and water purification. Additionally, MOFs might be used in sectors including gas storage, chemical sensing, and catalysis to improve the efficiency and sustainability of processes.

There have been encouraging findings on the use of MOFs in drug loading and delivery systems, particularly for antiviral and anticancer compounds. Future studies will concentrate on increasing the spectrum of pharmacological compounds that can be encapsulated within MOFs, enabling targeted administration and controlled release of a greater variety of therapeutic agents. Drug delivery methods may be completely changed as a result, increasing the effectiveness of treatments. Furthermore, MOFs show exciting promise in the field of biosensing. Its unique structure, tunability, and versatility make it an ideal material for efficient and sensitive biosensors. Toxicological research and commercialization to ensure the safety of MOFs for human usage, thorough toxicological research will be necessary as the technology moves closer to commercialization. Through continuous research and innovation, we can foresee that MOFs will bring revolutionary changes in biomedical research, clinical diagnosis, and treatment, and make important contributions to human health and medical progress.

Despite many efforts that have been invested in signal transduction technologies using MOFs, the huge challenges associated with the sensing implementation of MOFs toward signal transduction manners are still found due to the diversity of detection necessity. Hence, ultrasensitive signal transduction protocols are required to maintain high standards of medical diagnostics, food quality, and environmental safety. The non‐toxic MOF crystals with carefully selected ingredients that can result in high porosity and high thermal and chemical stability can be made to improve the functionality of future TENG‐based implantable devices and biodegradable sensors. Furthermore, the combination of the exciting features of MOFs and artificial technologies (AI) would promote the development of biomedical and healthcare applications ranging from wearable sensors to exoskeletons, electronic skin, etc.

In conclusion, the development of these materials for specialized applications, such as electrical devices, medication delivery, energy storage, environmental clean‐up, and industrial processes, will be fuelled by advances in our understanding of their structure, stability, and characteristics. Hence, MOFs have a bright future in a variety of industries. In the future, safer and more environmentally friendly goods will be commercialized thanks to the successful use of MOFs, which may be achieved by filling in the information gaps currently present and carrying out thorough toxicological assessments.

## Conflict of Interest

The authors declare no conflict of interest.

## References

[1] O. M. Yaghi , M. O'Keeffe , N. W. Ockwig , H. K. Chae , M. Eddaoudi , J. Kim , Nature 2003, 423, 705. 12802325 10.1038/nature01650

[2] H. Furukawa , K. E. Cordova , M. O'Keeffe , O. M. Yaghi , Science 2013, 341, 1230444. 23990564 10.1126/science.1230444

[3] H. C. Zhou , J. R. Long , O. M. Yaghi , Chem. Rev. 2012, 112, 673. 22280456 10.1021/cr300014x

[4] B. Li , H. M. Wen , H. L. Wang , H. Wu , M. Tyagi , T. Yildirim , W. Zhou , B. L. Chen , J. Am. Chem. Soc. 2014, 136, 6207. 24730649 10.1021/ja501810r

[5] H. Daglar , H. C. Gulbalkan , G. Avci , G. O. Aksu , O. F. Altundal , C. Altintas , I. Erucar , S. Keskin , Angew. Chem.‐Int. Ed. 2021, 60, 7828. 10.1002/anie.202015250 PMC8049020 33443312

[6] Y. Cui , Y. Yue , G. Qian , B. Chen , Chem. Rev. 2012, 112, 1126. 21688849 10.1021/cr200101d

[7] Y. F. Zhao , H. Zeng , X. W. Zhu , W. G. Lu , D. Li , Chem. Soc. Rev. 2021, 50, 4484. 33595006 10.1039/d0cs00955e

[8] J. Dong , D. Zhao , Y. Lu , W. Y. Sun , J. Mater. Chem. A 2019, 7, 22744.

[9] G. M. Espallargas , E. Coronado , Chem. Soc. Rev. 2018, 47, 533. 29112210 10.1039/c7cs00653e

[10] A. E. Thorarinsdottir , T. D. Harris , Chem. Rev. 2020, 120, 8716. 32045215 10.1021/acs.chemrev.9b00666

[11] A. Bavykina , N. Kolobov , I. S. Khan , J. A. Bau , A. Ramirez , J. Gascon , Chem. Rev. 2020, 120, 8468. 32223183 10.1021/acs.chemrev.9b00685

[12] V. Pascanu , G. G. Miera , A. K. Inge , B. Martin‐Matute , J. Am. Chem. Soc. 2019, 141, 7223. 30974060 10.1021/jacs.9b00733

[13] H. Tsai , S. Shrestha , R. A. Vila , W. X. Huang , C. M. Liu , C. H. Hou , H. H. Huang , X. W. Wen , M. X. Li , G. Wiederrecht , Y. Cui , M. Cotlet , X. Y. Zhang , X. D. Ma , W. Y. Nie , Nat. Photonics 2021, 15, 843.

[14] M. Gutierrez , C. Martin , K. Kennes , J. Hofkens , M. Vander Auweraer , F. Sanchez , A. Douhal , Adv. Opt. Mater. 2018, 6, 1701060.

[15] H. D. Lawson , S. P. Walton , C. Chan , ACS Appl. Mater. Interfaces 2021, 13, 7004. 33554591 10.1021/acsami.1c01089 PMC11790311

[16] J. L. Yang , H. Wang , J. Y. Liu , M. K. Ding , X. J. Xie , X. Y. Yang , Y. R. Peng , S. Zhou , R. Z. Ouyang , Y. Q. Miao , RSC Adv. 2021, 11, 3241. 35424280 10.1039/d0ra09878g PMC8694185

[17] Y. Liu , T. Jiang , Z. Liu , Nanotheranostics 2022, 6, 143. 34976590 10.7150/ntno.63458 PMC8671950

[18] H. S. Wang , Y. H. Wang , Y. Ding , Nanoscale Adv. 2020, 2, 3788. 36132764 10.1039/d0na00557f PMC9418943

[19] L. S. Xie , G. Skorupskii , M. Dinca , Chem. Rev. 2020, 120, 8536. 32275412 10.1021/acs.chemrev.9b00766 PMC7453401

[20] C. Li , L. L. Zhang , J. Q. Chen , X. L. Li , J. W. Sun , J. W. Zhu , X. Wang , Y. S. Fu , Nanoscale 2021, 13, 485. 33404574 10.1039/d0nr06396g

[21] B. He , Q. C. Zhang , Z. H. Pan , L. Li , C. W. Li , Y. Ling , Z. X. Wang , M. X. Chen , Z. Wang , Y. G. Yao , Q. W. Li , L. T. Sun , J. Wang , L. Wei , Chem. Rev. 2022, 122, 10087. 35446541 10.1021/acs.chemrev.1c00978 PMC9185689

[22] I. Hussain , S. Iqbal , C. Lamiel , A. Alfantazi , K. L. Zhang , J. Mater. Chem. A 2022, 10, 4475.

[23] S. Radhakrishnan , M. Mathew , C. S. Rout , Mater. Adv. 2022, 3, 1874.

[24] M. Daniel , G. Mathew , M. Anpo , B. Neppolian , Coord. Chem. Rev. 2022, 468, 214627.

[25] H. Zhou , Z. Ren , C. Xu , L. Xu , C. Lee , Nanomicro Lett 2022, 14, 207. 36271989 10.1007/s40820-022-00950-1 PMC9588146

[26] H. Zhou , X. D. Hui , D. X. Li , D. L. Hu , X. Chen , X. M. He , L. X. Gao , H. Huang , C. Lee , X. J. Mu , Adv. Sci. 2020, 7, 2001173. 10.1002/advs.202001173 PMC7578855 33101855

[27] Y. Q. Li , T. Ben , B. Y. Zhang , Y. Fu , S. L. Qiu , Scient. Rep. 2013, 3, 2420. 10.1038/srep02420 PMC3741621 23939301

[28] X. Y. Li , C. M. Zeng , J. Jiang , L. H. Ai , J. Mater. Chem. A 2016, 4, 7476.

[29] O. M. Yaghi , G. M. Li , H. L. Li , Nature 1995, 378, 703.

[30] H. Li , M. Eddaoudi , T. L. Groy , O. M. Yaghi , J. Am. Chem. Soc. 1998, 120, 8571.

[31] Z. H. Mai , D. X. Liu , Cryst. Growth Des. 2019, 19, 7439.

[32] J.‐R. Li , J. Sculley , H.‐C. Zhou , Chem. Rev. 2012, 112, 869. 21978134 10.1021/cr200190s

[33] C. E. Wilmer , M. Leaf , C. Y. Lee , O. K. Farha , B. G. Hauser , J. T. Hupp , R. Q. Snurr , Nat. Chem. 2012, 4, 83. 10.1038/nchem.1192 22270624

[34] S. Kaskel , The Chemistry of metal‐organic frameworks: synthesis, characterization, and applications, 1, John Wiley and Sons, Hoboken 2016.

[35] K. O. Kirlikovali , S. L. Hanna , F. A. Son , O. K. Farha , ACS Nanosc. Au 2022, 3, 37. 10.1021/acsnanoscienceau.2c00046 PMC10125349 37101466

[36] J. Wang , Y. L. Wang , H. B. Hu , Q. P. Yang , J. J. Cai , Nanoscale 2020, 12, 4238. 32039421 10.1039/c9nr09697c

[37] M. J. Huangfu , M. Wang , C. Lin , J. Wang , P. Y. Wu , Dalton Trans. 2021, 50, 3429. 33650595 10.1039/d0dt04276e

[38] G. L. Yang , X. L. Jiang , H. Xu , B. Zhao , Small 2021, 17, 2005327. 10.1002/smll.202005327 33634574

[39] Y. Li , Y. T. Wang , W. D. Fan , D. F. Sun , Dalton Trans. 2022, 51, 4608. 35225319 10.1039/d1dt03842g

[40] Y. Li , D. Gan , X. Deng , L. Jiang , C. Xie , X. Lu , Biosurf. Biotribol. 2022, 8, 151.

[41] B. Gibbons , M. Cai , A. J. Morris , J. Am. Chem. Soc. 2022, 144, 17723. 36126182 10.1021/jacs.2c04144 PMC9545145

[42] D. J. Cerasale , D. C. Ward , T. L. Easun , Nature Rev. Chem. 2022, 6, 9. 37117616 10.1038/s41570-021-00336-8

[43] Q. Wang , D. Astruc , Chem. Rev. 2020, 120, 1438. 31246430 10.1021/acs.chemrev.9b00223

[44] M. Zhong , L. J. Kong , K. Zhao , Y. H. Zhang , N. Li , X. H. Bu , Adv. Sci. 2021, 8, 2001980. 10.1002/advs.202001980 PMC7887588 33643787

[45] J. Yang , D. H. Dai , X. Zhang , L. S. Teng , L. J. Ma , Y. W. Yang , Theranostics 2023, 13, 295. 36593957 10.7150/thno.80687 PMC9800740

[46] A. E. Baumann , D. A. Burns , B. Q. Liu , V. S. Thoi , Commun. Chem. 2019, 2, 86.

[47] A. Zuliani , N. Khiar , C. Carrillo‐Carrion , Anal. Bioanal. Chem. 2023, 415, 2005. 36598537 10.1007/s00216-022-04493-7 PMC9811896

[48] H. Kaur , S. Sundriyal , V. Pachauri , S. Ingebrandt , K.‐H. Kim , A. L. Sharma , A. Deep , Coord. Chem. Rev. 2019, 401, 213077.

[49] X. Xiao , L. L. Zou , H. Pang , Q. Xu , Chem. Soc. Rev. 2020, 49, 301. 31832631 10.1039/c7cs00614d

[50] G. Khandelwal , A. Chandrasekhar , N. Raj , S. J. Kim , Adv. Energy Mater. 2019, 9, 1803581.

[51] Q. F. Shi , Z. X. Zhang , T. Chen , C. K. Lee , Nano Energy 2019, 62, 355.

[52] G. Khandelwal , N. Raj , S. J. Kim , Adv. Funct. Mater. 2020, 30, 1910162.

[53] G. Khandelwal , N. Raj , S. J. Kim , J. Mater. Chem. A 2020, 8, 17817.

[54] R. M. Wen , J. M. Guo , A. F. Yu , J. Y. Zhai , Z. L. Wang , Adv. Funct. Mater. 2019, 29, 1807655.

[55] Y. B. Guo , Y. L. Cao , Z. X. Chen , R. Li , W. Gong , W. F. Yang , Q. H. Zhang , H. Z. Wang , Nano Energy 2020, 70, 104517.

[56] G. Khandelwal , N. Raj , V. Vivekananthan , S. J. Kim , Iscience 2021, 24, 102064. 33554068 10.1016/j.isci.2021.102064 PMC7859291

[57] C. McKinstry , E. J. Cussen , A. J. Fletcher , S. V. Patwardhan , J. Sefcik , Cryst. Growth Des. 2013, 13, 5481.

[58] C. G. Lin , W. Zhou , X. T. Xiong , W. M. Xuan , P. J. Kitson , D. L. Long , W. Chen , Y. F. Song , L. Cronin , Angew. Chem. Int. Ed. 2018, 57, 16716. 10.1002/anie.201810095 PMC6391986 30370977

[59] Y. G. Xu , W. Liu , D. X. Li , H. H. Chen , M. Lu , Dalton Trans. 2017, 46, 11046. 28782787 10.1039/c7dt02582c

[60] Z. G. Hu , Y. X. Wang , D. Zhao , Accounts of Materials Research 2022, 3, 1106.

[61] Y. T. Qian , F. F. Zhang , H. Pang , Adv. Funct. Mater. 2021, 31, 2104231.

[62] J. F. Qian , F. A. Sun , L. Z. Qin , Mater. Lett. 2012, 82, 220.

[63] S. B. Novakovic , G. A. Bogdanovic , C. Heering , G. Makhloufi , D. Francuski , C. Janiak , Inorg. Chem. 2015, 54, 2660. 25706331 10.1021/ic5028256

[64] P. Railey , Y. Song , T. Y. Liu , Y. Li , Mater. Res. Bull. 2017, 96, 385.

[65] R. J. Kuppler , D. J. Timmons , Q. R. Fang , J. R. Li , T. A. Makal , M. D. Young , D. Q. Yuan , D. Zhao , W. J. Zhuang , H. C. Zhou , Coord. Chem. Rev. 2009, 253, 3042.

[66] V. F. Yusuf , N. I. Malek , S. K. Kailasa , ACS Omega 2022, 7, 44507. 36530292 10.1021/acsomega.2c05310 PMC9753116

[67] L. Xu , E. Y. Choi , Y. U. Kwon , Inorg. Chem. Commun. 2008, 11, 1190.

[68] E. R. Parnham , R. E. Morris , Acc. Chem. Res. 2007, 40, 1005. 17580979 10.1021/ar700025k

[69] J. B. Shi , J. L. Zhang , D. X. Tan , X. Y. Cheng , X. N. Tan , B. X. Zhang , B. X. Han , L. F. Liu , F. Y. Zhang , M. R. Liu , J. F. Xiang , ChemCatChem 2019, 11, 2058.

[70] S. Yuan , W. G. Lu , Y. P. Chen , Q. Zhang , T. F. Liu , D. W. Feng , X. Wang , J. S. Qin , H. C. Zhou , J. Am. Chem. Soc. 2015, 137, 3177. 25714137 10.1021/ja512762r

[71] J. Klinowski , F. A. A. Paz , P. Silva , J. Rocha , Dalton Trans. 2011, 40, 321. 20963251 10.1039/c0dt00708k

[72] V. N. Le , H. T. Kwon , T. K. Vo , J. H. Kim , W. S. Kim , J. Kim , Mater. Chem. Phys. 2020, 253, 123278.

[73] J. S. Choi , W. J. Son , J. Kim , W. S. Ahn , Microporous Mesoporous Mater. 2008, 116, 727.

[74] J. Hafizovic , M. Bjorgen , U. Olsbye , P. D. C. Dietzel , S. Bordiga , C. Prestipino , C. Lamberti , K. P. Lillerud , J. Am. Chem. Soc. 2007, 129, 3612. 17341071 10.1021/ja0675447

[75] P. P. Bag , X. S. Wang , R. Cao , Dalton Trans. 2015, 44, 11954. 26067212 10.1039/c5dt01598g

[76] P. J. Beldon , L. Fabian , R. S. Stein , A. Thirumurugan , A. K. Cheetham , T. Friscic , Angew. Chem. Int. Ed. 2010, 49, 9640. 10.1002/anie.201005547 21077080

[77] M. Klimakow , P. Klobes , A. F. Thunemann , K. Rademann , F. Emmerling , Chem. Mater. 2010, 22, 5216.

[78] A. Krusenbaum , S. Grätz , G. T. Tigineh , L. Borchardt , J. G. Kim , Chem. Soc. Rev. 2022, 51, 2873. 35302564 10.1039/d1cs01093j PMC8978534

[79] S. Glowniak , B. Szczesniak , J. Choma , M. Jaroniec , Mater. Today 2021, 46, 109. 10.1002/adma.202103477 34580939

[80] Y. Li , G. L. Wen , J. Z. Li , Q. R. Li , H. X. Zhang , B. Tao , J. Z. Zhang , Chem. Commun. (Camb.) 2022, 58, 11488. 36165339 10.1039/d2cc04190a

[81] A. M. Joaristi , J. Juan‐Alcaniz , P. Serra‐Crespo , F. Kapteijn , J. Gascon , Cryst. Growth Des. 2012, 12, 3489.

[82] T. R. C. Van Assche , G. Desmet , R. Ameloot , D. E. De Vos , H. Terryn , J. F. M. Denayer , Microp. Mesop. Mater. 2012, 158, 209.

[83] S. Sachdeva , A. Pustovarenko , E. J. R. Sudholter , F. Kapteijn , L. de Smet , J. Gascon , CrystEngComm 2016, 18, 4018.

[84] S. Glowniak , B. Szczesniak , J. Choma , M. Jaroniec , Molecules 2023, 28, 2639. 36985612 10.3390/molecules28062639 PMC10051140

[85] L. G. Qiu , Z. Q. Li , Y. Wu , W. Wang , T. Xu , X. Jiang , Chem. Commun. (Camb.) 2008, 3642. 18665285 10.1039/b804126a

[86] Z. Q. Li , L. G. Qiu , T. Xu , Y. Wu , W. Wang , Z. Y. Wu , X. Jiang , Mater. Lett. 2009, 63, 78.

[87] W. J. Son , J. Kim , J. Kim , W. S. Ahn , Chem. Commun. (Camb.) 2008, 47, 6336. 10.1039/b814740j 19048147

[88] N. A. Khan , S. H. Jhung , Coord. Chem. Rev. 2015, 285, 11.

[89] B. Q. Yao , S. K. Lua , H. S. Lim , Q. Zhang , X. Y. Cui , T. J. White , V. P. Ting , Z. L. Dong , Microporous Mesoporous Mater. 2021, 314, 110777.

[90] X. Wang , W. X. Chen , L. Zhang , T. Yao , W. Liu , Y. Lin , H. X. Ju , J. C. Dong , L. R. Zheng , W. S. Yan , X. S. Zheng , Z. J. Li , X. Q. Wang , J. Yang , D. S. He , Y. Wang , Z. X. Deng , Y. E. Wu , Y. D. Li , J. Am. Chem. Soc. 2017, 139, 9419. 28661130 10.1021/jacs.7b01686

[91] M. Kalaj , S. M. Cohen , ACS Central Science 2020, 6, 1046. 32724840 10.1021/acscentsci.0c00690 PMC7379093

[92] S. Mandal , S. Natarajan , P. Mani , A. Pankajakshan , Adv. Funct. Mater. 2021, 31, 2006291.

[93] W. T. Kou , C. X. Yang , X. P. Yan , J. Mater. Chem. A 2018, 6, 17861.

[94] A. M. Bumstead , I. Pakamore , K. D. Richards , M. F. Thorne , S. S. Boyadjieva , C. Castillo‐Blas , L. N. McHugh , A. F. Sapnik , D. S. Keeble , D. A. Keen , R. C. Evans , R. S. Forgan , T. D. Bennett , Chem. Mater. 2022, 34, 2187. 35578693 10.1021/acs.chemmater.1c03820 PMC9100367

[95] C. Y. Zhu , Y. Peng , W. S. Yang , Green Chemical Engineering 2021, 2, 17.

[96] I. G. Koryakina , S. V. Bachinin , E. N. Gerasimova , M. V. Timofeeva , S. A. Shipilovskikh , A. S. Bukatin , A. Sakhatskii , A. S. Timin , V. A. Milichko , M. V. Zyuzin , Chem. Eng. J. 2023, 452, 139450.

[97] C. Echaide‐Gorriz , C. Clement , F. Cacho‐Bailo , C. Tellez , J. Coronas , J. Mater. Chem. A 2018, 6, 5485.

[98] C. P. Mao , S. C. Wang , J. K. Li , Z. W. Feng , T. Zhang , R. Wang , C. H. Fan , X. Y. Jiang , ACS Nano 2023, 17, 2840. 36728704 10.1021/acsnano.2c11241

[99] A. Bendre , M. P. Bhat , K. H. Lee , T. Altalhi , M. A. Alruqi , M. Kurkuri , Mater. Tod. Adv. 2022, 13, 100205.

[100] S. Soni , P. K. Bajpai , C. Arora , Character. Appl. Nanomater. 2020, 3, 87.

[101] L. Liang , C. Liu , F. Jiang , Q. Chen , L. Zhang , H. Xue , H. L. Jiang , J. Qian , D. Yuan , M. Hong , Nat. Commun. 2017, 8, 1233. 29089480 10.1038/s41467-017-01166-3 PMC5663901

[102] O. Shekhah , Y. Belmabkhout , Z. J. Chen , V. Guillerm , A. Cairns , K. Adil , M. Eddaoudi , Nat. Commun. 2014, 5, 4288. 24964404 10.1038/ncomms5228 PMC4083436

[103] T. T. H. Ta , N. T. Pham , S. Do Ngoc , J VNU J. Sci. Math. Phys. 2016, 32, 67.

[104] O. K. Farha , A. O. Yazaydin , I. Eryazici , C. D. Malliakas , B. G. Hauser , M. G. Kanatzidis , S. T. Nguyen , R. Q. Snurr , J. T. Hupp , Nat. Chem. 2010, 2, 944. 20966950 10.1038/nchem.834

[105] A. G. Wong‐Foy , A. J. Matzger , O. M. Yaghi , J. Am. Chem. Soc. 2006, 128, 3494. 16536503 10.1021/ja058213h

[106] H. Furukawa , N. Ko , Y. B. Go , N. Aratani , S. B. Choi , E. Choi , A. O. Yazaydin , R. Q. Snurr , M. O'Keeffe , J. Kim , O. M. Yaghi , Science 2010, 329, 424. 20595583 10.1126/science.1192160

[107] K. Sumida , D. L. Rogow , J. A. Mason , T. M. McDonald , E. D. Bloch , Z. R. Herm , T. H. Bae , J. R. Long , Chem. Rev. 2012, 112, 724. 22204561 10.1021/cr2003272

[108] J. M. Yu , L. H. Xie , J. R. Li , Y. G. Ma , J. M. Seminario , P. B. Balbuena , Chem. Rev. 2017, 117, 9674. 28394578 10.1021/acs.chemrev.6b00626

[109] T. Ghanbari , F. Abnisa , W. Daud , Sci. Total Environ. 2020, 707. 10.1016/j.scitotenv.2019.135090 31863992

[110] J. An , S. J. Geib , N. L. Rosi , J. Am. Chem. Soc. 2010, 132, 38. 20000664 10.1021/ja909169x

[111] D. Yan , B. L. Chen , Q. Duan , Inorg. Chem. Commun. 2014, 49, 34.

[112] H. Lyu , O. I. F. Chen , N. Hanikel , M. I. Hossain , R. W. Flaig , X. K. Pei , A. Amin , M. D. Doherty , R. K. Impastato , T. G. Glover , D. R. Moore , O. M. Yaghi , J. Am. Chem. Soc. 2022, 144, 2387. 35080872 10.1021/jacs.1c13368

[113] H. Ji , K. Naveen , W. Lee , T. S. Kim , D. Kim , D. H. Cho , ACS Appl. Mater. Interfaces 2020, 12, 24868. 32394698 10.1021/acsami.0c05912

[114] S. M. Cohen , Chem. Rev. 2012, 112, 970. 21916418 10.1021/cr200179u

[115] X. B. Wang , P. X. Zhang , Z. Q. Zhang , L. F. Yang , Q. Ding , X. L. Cui , J. Wang , H. B. Xing , Ind. Eng. Chem. Res. 2020, 59, 3531.

[116] J. Z. Wang , X. P. Fu , Q. Y. Liu , L. Chen , L. P. Xu , Y. L. Wang , Inorg. Chem. 2022, 61, 5737. 35385262 10.1021/acs.inorgchem.1c03740

[117] Z. T. Lin , Q. Y. Liu , L. Yang , C. T. He , L. Li , Y. L. Wang , Inorg. Chem. 2020, 59, 4030. 32118410 10.1021/acs.inorgchem.0c00003

[118] T. Ke , Q. J. Wang , J. Shen , J. Y. Zhou , Z. B. Bao , Q. W. Yang , Q. L. Ren , Angew. Chem. Int. Ed. 2020, 59, 12725. 10.1002/anie.202003421 32329164

[119] L. B. Li , R. B. Lin , R. Krishna , H. Li , S. C. Xiang , H. Wu , J. P. Li , W. Zhou , B. L. Chen , Science 2018, 362, 443. 30361370 10.1126/science.aat0586

[120] K. Shao , H. M. Wen , C. C. Liang , X. Y. Xiao , X. W. Gu , B. L. Chen , G. D. Qian , B. Li , Angew. Chem. Int. Ed. 2022, 61, e202211523. 10.1002/anie.202211523 35979632

[121] R. B. Lin , S. C. Xiang , W. Zhou , B. L. Chen , Chem 2020, 6, 337.

[122] K. Adil , Y. Belmabkhout , R. S. Pillai , A. Cadiau , P. M. Bhatt , A. H. Assen , G. Maurin , M. Eddaoudi , Chem. Soc. Rev. 2017, 46, 3402. 28555216 10.1039/c7cs00153c

[123] B. Li , H. M. Wen , W. Zhou , B. L. Chen , J. Phys. Chem. Lett. 2014, 5, 3468. 26278595 10.1021/jz501586e

[124] C. Altintas , O. F. Altundal , S. Keskin , R. Yildirim , J. Chem. Inf. Model. 2021, 61, 2131. 33914526 10.1021/acs.jcim.1c00191 PMC8154255

[125] B. Wang , L. H. Xie , X. Q. Wang , X. M. Liu , J. P. Li , J. R. Li , Green Energ. Env. 2018, 3, 191.

[126] H. Wang , M. Warren , J. Jagiello , S. Jensen , S. K. Ghose , K. Tan , L. Yu , T. J. Emge , T. Thonhauser , J. Li , J. Am. Chem. Soc. 2020, 142, 20088. 33172264 10.1021/jacs.0c09475

[127] O. Shekhah , Y. Belmabkhout , Z. J. Chen , V. Guillerm , A. Cairns , K. Adil , M. Eddaoudi , Nat. Commun. 2014, 5, 4228. 24964404 10.1038/ncomms5228 PMC4083436

[128] C. Simms , A. Mullaliu , S. Swinnen , F. de Azambuja , T. N. Parac‐Vogt , Molecul. Syst. Design Eng. 2023, 8, 270.

[129] H. F. Zheng , Y. J. Fan , A. L. Blenko , W. B. Lin , J. Am. Chem. Soc. 2023, 145, 9994. 37125994 10.1021/jacs.3c02703

[130] J. Xu , H. Shimakoshi , Y. Hisaeda , J. Organomet. Chem. 2015, 782, 89.

[131] J. S. Qin , D. Y. Du , W. Guan , X. J. Bo , Y. F. Li , L. P. Guo , Z. M. Su , Y. Y. Wang , Y. Q. Lan , H. C. Zhou , J. Am. Chem. Soc. 2015, 137, 7169. 25933041 10.1021/jacs.5b02688

[132] J. X. Qin , J. Wang , J. J. Yang , Y. Hu , M. L. Fu , D. Q. Ye , Appl. Catalysis B‐Env. 2020, 267, 118667.

[133] X. Li , S. W. Wang , X. Zhang , D. H. Mei , Y. H. Xu , P. Yu , Y. J. Sun , J. Clean. Prod. 2022, 332, 130107.

[134] T. A. Goetjen , J. Liu , Y. F. Wu , J. Y. Sui , X. Zhang , J. T. Hupp , O. K. Farha , Chem. Commun. (Camb.) 2020, 56, 10409. 32745156 10.1039/d0cc03790g

[135] K. K. Gangu , S. B. Jonnalagadda , Front. Chem. 2021, 9. 10.3389/fchem.2021.747615 PMC8718437 34976945

[136] Y. Y. Zhang , G. L. Lu , D. F. Zhao , X. B. Huang , Mater. Chem. Front. 2023, 7, 4782.

[137] M. Kurmoo , Chem. Soc. Rev. 2009, 38, 1353. 19384442 10.1039/b804757j

[138] E. Coronado , G. M. Espallargas , Chem. Soc. Rev. 2013, 42, 1525. 23146915 10.1039/c2cs35278h

[139] C. N. R. Rao , S. Natarajan , R. Vaidhyanathan , Angew. Chem. Int. Ed. 2004, 43, 1466. 10.1002/anie.200300588 15022215

[140] Q. Yu , D. Wang , J. Mater. Chem. A 2023, 11, 5548.

[141] N. Guillou , C. Livage , M. Drillon , G. Ferey , Angew. Chem. Int. Ed. 2003, 42, 5314. 10.1002/anie.200352520 14613163

[142] M. Chen , H. Zhao , Z. W. Wang , E. C. Sanudo , C. S. Liu , Inorg. Chem. Commun. 2015, 56, 48.

[143] S. Muratovic , V. Martinez , B. Karadeniz , D. Pajic , I. Brekalo , M. Arhangelskis , M. Mazaj , G. Mali , M. Etter , T. Friscic , Y. Krupskaya , V. Kataev , D. Zilic , K. Uzarevic , Inorg. Chem. 2022, 61, 18181. 36318217 10.1021/acs.inorgchem.2c02898

[144] X. M. M. Wang , H. X. Kou , J. Wang , R. Teng , X. Z. Du , X. Q. Lu , J. Porous Mater. 2020, 27, 1171.

[145] A. N. Zhu , T. Xuan , Y. Zhai , Y. P. Wu , X. Y. Guo , Y. Ying , Y. Wen , H. F. Yang , Sens. Actuators. B‐Chemical 2021, 339, 129909.

[146] M. Orts‐Arroyo , R. Rabelo , A. Carrasco‐Berlanga , N. Moliner , J. Cano , M. Julve , F. Lloret , G. De Munno , R. Ruiz‐Garcia , J. Mayans , J. Martinez‐Lillo , I. Castro , Dalton Trans. 2021, 50, 3801. 33721007 10.1039/d1dt00462j

[147] K. A. Gschneidner , V. K. Pecharsky , A. O. Tsokol , Rep. Prog. Phys. 2005, 68, 1479.

[148] Y. Tang , H. L. Wu , W. Q. Cao , Y. J. Cui , G. D. Qian , Adv. Opt. Mater. 2021, 9, 2001817.

[149] N. Armaroli , V. Balzani , Energ. Env. Sci. 2011, 4, 3193.

[150] J. Chang , ECS J. Solid State Sci. Technol. 2021, 10, 086009.

[151] L. N. Xu , Y. N. Li , Q. J. Pan , D. Wang , S. J. Li , G. F. Wang , Y. J. Chen , P. F. Zhu , W. P. Qin , ACS Appl. Mater. Interfaces 2020, 12, 18934. 32233390 10.1021/acsami.0c02999

[152] J. Y. Wang , M. H. Tai , Z. K. Yu , S. Kang , D. L. Jin , L. C. Wang , Dalton Trans. 2023, 52, 1212. 36645320 10.1039/d2dt03654a

[153] H. Li , H. B. Liu , X. M. Tao , J. Su , P. F. Ning , X. F. Xu , Y. Zhou , W. Gu , X. Liu , Dalton Trans. 2018, 47, 8427. 29897073 10.1039/c8dt01477a

[154] H. Y. Yu , J. C. Liu , S. Bao , G. Y. Gao , H. Y. Zhu , P. F. Zhu , G. F. Wang , Chem. Eng. J. 2022, 430, 132782.

[155] Q. Zhang , W. Y. Ge , X. M. Zhang , X. L. Chen , Dalton Trans. 2022, 51, 8714. 35611935 10.1039/d2dt00979j

[156] J. J. Ren , A. Meijerink , X. P. Zhou , J. P. Wu , G. Y. Zhang , Y. H. Wang , ACS Appl. Mater. Interfaces 2022, 14, 3176. 34981922 10.1021/acsami.1c20804

[157] G. Haider , M. Usman , T. P. Chen , P. Perumal , K. L. Lu , Y. F. Chen , ACS Nano 2016, 10, 8366. 27576847 10.1021/acsnano.6b03030

[158] M. Usman , G. Haider , S. Mendiratta , T. T. Luo , Y. F. Chen , K. L. Lu , J. Mater. Chem. C 2016, 4, 4728.

[159] Z. D. Lian , B. Wang , Z. S. Wu , H. Lin , S. S. Yan , J. L. Li , K. Zhang , Q. G. Zeng , J. C. Xu , S. Chen , S. P. Wang , K. W. Ng , ACS Appl. Nano Mater. 2023, 6, 1808.

[160] Y. Q. Shen , J. Ma , S. J. Li , J. J. Qian , Q. P. Li , Chem. Eng. J. 2023, 466, 143290.

[161] Y. Tang , H. L. Wu , W. Q. Cao , Y. J. Cui , G. D. Qian , Adv. Opt. Mater. 2021, 9, 2001817.

[162] A. C. McKinlay , R. E. Morris , P. Horcajada , G. Ferey , R. Gref , P. Couvreur , C. Serre , Angew. Chem. Int. Ed. 2010, 49, 6260. 10.1002/anie.201000048 20652915

[163] P. Horcajada , C. Serre , M. Vallet‐Regi , M. Sebban , F. Taulelle , G. Ferey , Angew. Chem. Int. Ed. 2006, 45, 5974. 10.1002/anie.200601878 16897793

[164] S. Yao , Y. T. Wang , J. J. Chi , Y. R. Yu , Y. J. Zhao , Y. Luo , Y. G. Wang , Adv. Sci. 2022, 9, e2103449. 10.1002/advs.202103449 PMC8787387 34783460

[165] J. L. Harding , J. M. Metz , M. M. Reynolds , Adv. Funct. Mater. 2014, 24, 7503.

[166] M. C. Bernini , D. Fairen‐Jimenez , M. Pasinetti , A. J. Ramirez‐Pastor , R. Q. Snurr , J. Mater. Chem. B 2014, 2, 766. 32261308 10.1039/c3tb21328e

[167] H. M. Wang , Y. Q. Chen , H. Wang , X. Q. Liu , X. Zhou , F. Wang , Angew. Chem. Int. Ed. 2019, 58, 7380. 10.1002/anie.201902714 30916460

[168] S. F. Yao , X. J. Zhao , L. Y. Wang , F. Chen , H. Gong , C. Y. Chen , C. Q. Cai , Microchim. Acta 2022, 189, 266. 10.1007/s00604-022-05340-3 35776208

[169] B. Tan , S. S. Zhang , K. M. Wang , Y. L. Yan , Z. L. Chu , Q. W. Wang , X. Li , G. F. Zhu , J. Fan , H. M. Zhao , Microchim. Acta 2022, 189, 295. 10.1007/s00604-022-05389-0 35882703

[170] B. Liu , L. R. Sun , X. J. Lu , Y. P. Yang , H. S. Peng , Z. G. Sun , J. Xu , H. Q. Chu , RSC Adv. 2022, 12, 11119. 35425048 10.1039/d1ra09320g PMC8992360

[171] K. Y. Ni , G. X. Lan , W. B. Lin , ACS Cent. Sci. 2020, 6, 861. 32607433 10.1021/acscentsci.0c00397 PMC7318063

[172] S. Keskin , S. Kizilel , Ind. Eng. Chem. Res. 2011, 50, 1799.

[173] D. Zhao , W. Zhang , Z. H. Wu , H. Xu , Front. Chem. 2022, 9, 834171. 35141208 10.3389/fchem.2021.834171 PMC8819150

[174] M. Pourmadadi , S. Ostovar , M. M. Eshaghi , M. Rajabzadeh‐Khosroshahi , S. Safakhah , S. Ghotekar , A. Rahdar , A. M. Diez‐Pascual , Appl. Organomet. Chem. 2023, 37, e6982.

[175] Q. Shi , Y. Yang , Z. Sun , C. Lee , ACS Mater. Au 2022, 2, 394. 36855708 10.1021/acsmaterialsau.2c00001 PMC9928409

[176] Z. Zhang , F. Wen , Z. Sun , X. Guo , T. He , C. Lee , Adv. Intell. Syst. 2022, 4, 2100228.

[177] L. Liu , X. Guo , C. Lee , Nano Energy 2021, 88, 106304.

[178] Z. Zhang , L. Wang , C. Lee , Adv. Sens. Res. 2023, 2, 2200072.

[179] Z. Sun , M. Zhu , C. Lee , Nanoenergy Adv. 2021, 1, 81.

[180] T. He , C. Lee , IEEE Open J. Circ. Syst. 2021, 2, 702.

[181] Y. Wang , Y. Yang , Z. L. Wang , npj Flex. Electron. 2017, 1, 10.

[182] S. M. Niu , S. H. Wang , L. Lin , Y. Liu , Y. S. Zhou , Y. F. Hu , Z. L. Wang , Energ. Env. Sci. 2013, 6, 3576.

[183] Y. M. Wang , X. X. Zhang , C. S. Liu , L. T. Wu , J. J. Zhang , T. M. Lei , Y. Wang , X. B. Yin , R. S. Yang , Nano Energy 2023, 107, 108149.

[184] Y. Yang , X. Guo , M. Zhu , Z. Sun , Z. Zhang , T. He , C. Lee , Adv. Energ. Mater. 2022, 13, 2203040.

[185] J. K. Chen , H. W. Guo , P. Ding , R. Z. Pan , W. B. Wang , W. P. Xuan , X. Z. Wang , H. Jin , S. R. Dong , J. K. Luo , Nano Energy 2016, 30, 235.

[186] P. Bai , G. Zhu , Y. S. Zhou , S. H. Wang , J. S. Ma , G. Zhang , Z. L. Wang , Nano Res. Res. 2014, 7, 990.

[187] S. H. Wang , L. Lin , Z. L. Wang , Nano Lett. 2012, 12, 6339. 23130843 10.1021/nl303573d

[188] S. S. Kwak , H. Kim , W. Seung , J. Kim , R. Hinchet , S. W. Kim , ACS Nano 2017, 11, 10733. 28968064 10.1021/acsnano.7b05203

[189] Z. L. Wang , ACS Nano 2013, 7, 9533. 24079963 10.1021/nn404614z

[190] G. Khandelwal , N. Raj , S. J. Kim , Adv. Energ. Mater. 2021, 11, 2101170.

[191] H. Li , M. Eddaoudi , M. O'Keeffe , O. M. Yaghi , Nature 1999, 402, 276.

[192] S. Nagappan , M. Duraivel , V. Elayappan , N. Muthuchamy , B. Mohan , A. Dhakshinamoorthy , K. Prabakar , J. M. Lee , K. H. Park , Energy Technol. Technol. 2023, 11, 2201200.

[193] J. Yan , T. Liu , X. D. Liu , Y. H. Yan , Y. Huang , Coord. Chem. Rev. 2022, 452, 214300.

[194] S. Sundriyal , H. Kaur , S. K. Bhardwaj , S. Mishra , K. H. Kim , A. Deep , Coord. Chem. Rev. 2018, 369, 15.

[195] S. Hajra , M. Sahu , A. M. Padhan , I. S. Lee , D. K. Yi , P. Alagarsamy , S. S. Nanda , H. J. Kim , Adv. Funct. Mater. 2021, 31, 2101829.

[196] C. Huang , G. Z. Lu , N. Qin , Z. C. Shao , D. B. Zhang , C. Soutis , Y. Y. Zhang , L. W. Mi , H. W. Hou , ACS Appl. Mater. Interfaces 2022, 14, 16424. 35377137 10.1021/acsami.2c01251

[197] M. Salauddin , S. S. Rana , M. Sharifuzzaman , S. H. Lee , M. Abu Zahed , Y. Do Shin , S. Seonu , H. S. Song , T. Bhatta , J. Y. Park , Nano Energy 2022, 100, 107462.

[198] S. Hajra , M. Sahu , R. Sahu , A. M. Padhan , P. Alagarsamy , H. G. Kim , H. Lee , S. Oh , Y. Yamauchi , H. J. Kim , Nano Energy 2022, 98, 107253.

[199] Y. M. Wang , X. X. Zhang , D. Y. Yang , L. T. Wu , J. J. Zhang , T. M. Lei , R. Yang , Nanotechnology 2022, 33, 065402.

[200] Y. J. Hu , Y. L. Wang , S. D. Tian , A. F. Yu , L. Y. Wan , J. Y. Zhai , Macromol. Mater. Eng. 2021, 306, 2100128.

[201] J. Park , M. Lee , D. W. Feng , Z. H. Huang , A. C. Hinckley , A. Yakoyenko , X. D. Zou , Y. Cui , Z. A. Bao , J. Am. Chem. Soc. 2018, 140, 10315. 30041519 10.1021/jacs.8b06020

[202] Y. Yan , P. Gu , S. S. Zheng , M. B. Zheng , H. Pang , H. G. Xue , J. Mater. Chem. A 2016, 4, 19078.

[203] C. Y. Yang , X. Y. Li , L. Yu , X. J. Liu , J. Yang , M. D. Wei , Chem. Commun. (Camb.) 2020, 56, 1803. 31950124 10.1039/c9cc09302h

[204] D. D. Chen , L. S. Wei , J. F. Li , Q. S. Wu , J. Energy Storage. 2020, 30, 101525.

[205] D. Sheberla , J. C. Bachman , J. S. Elias , C. J. Sun , Y. Shao‐Horn , M. Dinca , Nat. Mater. 2017, 16, 220. 27723738 10.1038/nmat4766

[206] D. K. Nguyen , I. M. Schepisi , F. Z. Amir , Chem. Eng. J. 2019, 378, 122150.

[207] S. K. Shinde , D. Y. Kim , M. Kumar , G. Murugadoss , S. Ramesh , A. M. Tamboli , H. M. Yadav , Polymers 2022, 14, 511. 35160499 10.3390/polym14030511 PMC8839617

[208] S. Khokhar , H. Anand , P. Chand , J. Energy Storage. 2022, 56, 105897.

[209] R. Vinodh , R. S. Babu , S. Sambasivam , C. Gopi , S. Alzahmi , H. J. Kim , A. L. F. de Barros , I. M. Obaidat , Nanomaterials 2022, 12, 1511. 35564227 10.3390/nano12091511 PMC9105330

[210] K. Jayaramulu , M. Horn , A. Schneemann , H. Saini , A. Bakandritsos , V. Ranc , M. Petr , V. Stavila , C. Narayana , B. Scheibe , S. Kment , M. Otyepka , N. Motta , D. Dubal , R. Zboril , R. A. Fischer , Adv. Mater. 2021, 33, 2004560. 10.1002/adma.202004560 PMC11468759 33274794

[211] B. L. Wang , W. Li , Z. L. Liu , Y. J. Duan , B. Zhao , Y. Wang , J. H. Liu , RSC Adv. 2020, 10, 12129. 35497584 10.1039/c9ra10467d PMC9050789

[212] M. K. Mishra , V. Dubey , P. Mishra , I. J. J. o. E. R. Khan , Reports 2019, 4, 1.

[213] R. Bogue , Sensor Rev. 2013, 33, 300.

[214] K. J. Rebello , Proceedings IEEE 2004, 92, 43.

[215] D. L. Polla , A. G. Erdman , W. P. Robbins , D. T. Markus , J. Diaz‐Diaz , R. Rizq , Y. Nam , H. T. Brickner , A. Wang , P. Krulevitch , Annu. Rev. Biomed. Eng. 2000, 2, 551. 11701523 10.1146/annurev.bioeng.2.1.551

[216] S. Bhansali , A. Vasudev , MEMS for biomedical applications, Elsevier, Amsterdam 2012.

[217] J. A. Walker , J. Micromech. Microeng. 2000, 10, R1.

[218] S. Finkbeiner , presented at IEEE 43rd Conf. Eur. Solid‐State Device Res., Bucharest, ROMANIA, Sep 16–20, 2013.

[219] D. S. Eddy , D. R. Sparks , Proceed. IEEE 1998, 86, 1747.

[220] D. K. Shaeffer , IEEE Commun. Mag. 2013, 51, 100.

[221] W. P. Eaton , J. H. Smith , Smart Mater. Struct. 1997, 6, 530.

[222] D. M. Marom , D. T. Neilson , D. S. Greywall , C.‐S. Pai , N. R. Basavanhally , V. A. Aksyuk , D. O. López , F. Pardo , M. E. Simon , Y. J. J. o. L. T. Low , 2005, 23, 1620.

[223] K. Roy , J. E. Lee , C. Lee , Microsyst. Nanoeng. 2023, 9, 95. 37484500 10.1038/s41378-023-00555-7 PMC10359338

[224] A. Paramanick , K. Roy , D. Samanta , K. Aiswarya , R. Pratap , M. S. Singh , presented at Photons Plus Ultrasound Imaging Sens. 2023, 2023.

[225] K. Roy , H. Gupta , V. Shastri , A. Dangi , A. Jeyaseelan , S. Dutta , R. Pratap , IEEE Sens. J. 2020, 20, 6802.

[226] K. Roy , K. Kalyan , A. Ashok , V. Shastri , A. A. Jeyaseelan , A. Mandal , R. Pratap , J. Microelectromech. Syst. 2021, 30, 642.

[227] A. Dangi , K. Roy , S. Agrawal , H. Y. Chen , A. Ashok , C. Wible , M. Osman , R. Pratap , S. R. Kothapalli , presented at Conf. Photons Plus Ultrasound Imaging Sens. 2020, San Francisco, CA, Feb 02–05, 2020.

[228] K. Roy , V. Shastri , A. Kumar , J. Rout , Isha, K. Kalyan , J. Prakash , R. Pratap , presented at Conf. Photons Plus Ultrasound Imaging Sens., Electr Network, Jan 22‐Feb 24, 2022.

[229] K. Roy , A. Mandal , A. Ashok , H. Gupta , V. Shastri , R. Pratap , Ieee, presented at IEEE Int. Ultrasonics Symp. (IEEE IUS), Las Vegas, NV, Sep 07–11, 2020.

[230] K. Roy , K. Kalyan , A. Ashok , V. Shastri , R. Pratap , Ieee, presented at IEEE Int. Ultrasonics Symp. (IEEE IUS), Electr Network, Sep 11–16, 2021.

[231] K. Roy , K. Kalyan , A. Ashok , V. Shastri , R. Pratap , IEEE, presented at 21st Int.Conf. Solid‐State Sens., Actuat. Microsys. (Transducers), Electr Network, Jun 20–25, 2021.

[232] K. Roy , A. Ashok , K. Kalyan , V. Shastri , A. Jeyaseelan , M. Nayak , R. Pratap , presented at Conf. Photons Plus Ultrasound Imaging Sens. 2021, 2021.

[233] M. I. A. Asri , M. N. Hasan , M. R. A. Fuaad , Y. M. Yunos , M. S. M. Ali , IEEE Sens. J. 2021, 21, 18381.

[234] X. Yan , H. M. Qu , Y. Chang , W. Pang , Y. Y. Wang , X. X. Duan , ACS Appl. Mater. Interfaces 2020, 12, 10009. 31927971 10.1021/acsami.9b22407

[235] A. L. Robinson , V. L. Stavila , T. R. Zeitler , M. I. White , S. M. Thornberg , J. A. Greathouse , M. D. Allendorf , Anal. Chem. 2012, 84, 7043. 22905832 10.1021/ac301183w

[236] P. Davydovskaya , A. Ranft , B. V. Lotsch , R. Pohle , Anal. Chem. 2014, 86, 6948. 24939583 10.1021/ac500759n

[237] Z. H. Ma , T. W. Yuan , Y. Fan , L. Y. Wang , Z. M. Duan , W. Du , D. Zhang , J. Q. Xu , Sens. Actuators. B‐Chemical 2020, 311, 127365.

[238] S. R. Cai , W. Li , P. C. Xu , X. Y. Xia , H. T. Yu , S. Zhang , X. X. Li , Analyst 2019, 144, 3729. 30963147 10.1039/c8an02508h

[239] T. Xu , P. C. Xu , D. Zheng , H. T. Yu , X. X. Li , Anal. Chem. 2016, 88, 12234. 28193061 10.1021/acs.analchem.6b03364

[240] M. D. Allendorf , R. J. T. Houk , L. Andruszkiewicz , A. A. Talin , J. Pikarsky , A. Choudhury , K. A. Gall , P. J. Hesketh , J. Am. Chem. Soc. 2008, 130, 14404. 18841964 10.1021/ja805235k

[241] N. Jaber , S. Ilyas , O. Shekhah , M. Eddaoudi , M. I. Younis , Sens. Actuators. A‐Physical 2018, 283, 254.

[242] L. Y. Zhu , K. P. Yuan , Z. C. Li , X. Y. Miao , J. C. Wang , S. H. Sun , A. Devi , H. L. Lu , J. Colloid Interface Sci. 2022, 622, 156. 35490619 10.1016/j.jcis.2022.04.081

[243] M. R. Venkatesh , S. Sachdeva , B. El Mansouri , J. Wei , A. Bossche , D. Bosma , L. de Smet , E. J. R. Sudholter , G. Q. Zhang , Sensors 2019, 19, 888. 30791657 10.3390/s19040888 PMC6412504

[244] M. Ding , R. W. Flaig , H. L. Jiang , O. M. Yaghi , Chem. Soc. Rev. 2019, 48, 2783. 31032507 10.1039/c8cs00829a

[245] W. Cai , J. Wang , C. Chu , W. Chen , C. Wu , G. Liu , Adv. Sci. 2019, 6, 1801526. 10.1002/advs.201801526 PMC6325578 30643728

[246] X. Zhao , Y. Wang , D. S. Li , X. Bu , P. Feng , Adv. Mater. 2018, 30, 1705189. 10.1002/adma.201705189 29582482

[247] M. Woellner , S. Hausdorf , N. Klein , P. Mueller , M. W. Smith , S. Kaskel , Adv. Mater. 2018, 30, 1704679. 10.1002/adma.201704679 29921016

[248] K. S. Park , Z. Ni , A. P. Cote , J. Y. Choi , R. Huang , F. J. Uribe‐Romo , H. K. Chae , M. O'Keeffe , O. M. Yaghi , Proc Natl Acad Sci U S A 2006, 103, 10186. 16798880 10.1073/pnas.0602439103 PMC1502432

[249] D. W. Lim , H. Kitagawa , Chem. Soc. Rev. 2021, 50, 6349. 33870975 10.1039/d1cs00004g

[250] Q. L. Zhu , Q. Xu , Chem. Soc. Rev. 2014, 43, 5468. 24638055 10.1039/c3cs60472a

[251] I. Luz , M. Soukri , M. Lail , Chem. Sci. 2018, 9, 4589. 29899952 10.1039/c7sc05372j PMC5969507

[252] G. Cai , P. Yan , L. Zhang , H. C. Zhou , H. L. Jiang , Chem. Rev. 2021, 121, 12278. 34280313 10.1021/acs.chemrev.1c00243

[253] H. Zhou , D. Hu , C. Yang , C. Chen , J. Ji , M. Chen , Y. Chen , Y. Yang , X. Mu , Scient. Rep. 2018, 8, 14801. 10.1038/s41598-018-32827-y PMC6172240 30287826

[254] H. Zhou , D. Li , X. Hui , X. Mu , Int. J. Optomechatronics 2021, 15, 97.

[255] H. Zhou , D. Li , Z. Ren , X. Mu , C. Lee , InfoMat 2022, 4, e12349.

[256] H. Zhou , L. Xu , Z. Ren , J. Zhu , C. Lee , Nanoscale Adv. 2023, 5, 538. 36756499 10.1039/d2na00608a PMC9890940

[257] H. Zhou , C. Yang , D. Hu , S. Dou , X. Hui , F. Zhang , C. Chen , M. Chen , Y. Yang , X. Mu , ACS Appl. Mater. Interfaces 2019, 11, 14630. 30920795 10.1021/acsami.9b02087

[258] H. Zhou , C. Yang , D. Hu , D. Li , X. Hui , F. Zhang , M. Chen , X. Mu , Appl. Phys. Lett. 2019, 115, 143507.

[259] X. Hui , C. Yang , D. Li , X. He , H. Huang , H. Zhou , M. Chen , C. Lee , X. Mu , Adv. Sci. 2021, 8, e2100583. 10.1002/advs.202100583 PMC8373097 34155822

[260] D. Li , H. Zhou , Z. Chen , Z. Ren , C. Xu , X. He , T. Liu , X. Chen , H. Huang , C. Lee , X. Mu , Adv. Mater. 2023, 35, 2301787. 10.1002/adma.202301787 37204145

[261] D. Li , H. Zhou , X. Hui , X. He , X. Mu , Anal. Chem. 2021, 93, 9437. 34170680 10.1021/acs.analchem.1c01078 PMC8262173

[262] H. Zhou , Z. Ren , D. Li , C. Xu , X. Mu , C. Lee , Nat. Commun. 2023, 14. 37952033 10.1038/s41467-023-43127-z PMC10640644

[263] C. Xu , Z. Ren , H. Zhou , J. Zhou , C. P. Ho , N. Wang , C. Lee , Light Sci. Appl. 2023, 12, 154. 37357238 10.1038/s41377-023-01186-3 PMC10290984

[264] C. Xu , Z. Ren , J. Wei , C. Lee , iScience 2022, 25, 103799. 35198867 10.1016/j.isci.2022.103799 PMC8841618

[265] Z. Ren , Z. Zhang , J. Wei , B. Dong , C. Lee , Nat. Commun. 2022, 13, 3859. 35790752 10.1038/s41467-022-31520-z PMC9256719

[266] W. Liu , Y. Ma , X. Liu , J. Zhou , C. Xu , B. Dong , C. Lee , Nano Lett. 2022, 22, 6112. 35759415 10.1021/acs.nanolett.2c01198

[267] X. Chong , Y. Zhang , E. Li , K.‐J. Kim , P. R. Ohodnicki , C.‐h. Chang , A. X. Wang , ACS Sens. 2018, 3, 230. 29262684 10.1021/acssensors.7b00891

[268] P. Pashazadeh‐Panahi , S. Belali , H. Sohrabi , F. Oroojalian , M. Hashemzaei , A. Mokhtarzadeh , M. de la Guardia , Trac‐Trends Anal. Chem. 2021, 141, 116285.

[269] J. E. Cun , X. Fan , Q. Q. Pan , W. X. Gao , K. Luo , B. He , Y. J. Pu , Adv. Colloid Interface Sci. 2022, 305, 102686. 35523098 10.1016/j.cis.2022.102686

[270] G. S. Chen , S. M. Huang , X. X. Kou , S. B. Wei , S. Y. Huang , S. Q. Jiang , J. Shen , F. Zhu , G. F. Ouyang , Angew. Chem. Int. Ed. 2019, 58, 1463. 10.1002/anie.201813060 30536782

[271] J. J. Chen , Y. F. Zhu , S. Kaskel , Angew. Chem. Int. Ed. 2021, 60, 5010. 10.1002/anie.201909880 PMC7984248 31989749

[272] M. Z. Lv , W. Zhou , H. Tavakoli , C. Bautista , J. F. Xia , Z. H. Wang , X. J. Li , Biosens. Bioelectron. 2021, 176, 112947. 33412430 10.1016/j.bios.2020.112947 PMC7855766

[273] C. Jia , T. He , G. M. Wang , Coord. Chem. Rev. 2023, 476, 214930.

[274] H. S. Wang , Coord. Chem. Rev. 2017, 349, 139.

[275] S. A. Younis , N. Bhardwaj , S. K. Bhardwaj , K. H. Kim , A. Deep , Coord. Chem. Rev. 2021, 429, 213620.

[276] Y. Liu , X. Y. Xie , C. Cheng , Z. S. Shao , H. S. Wang , J. Mater. Chem. C 2019, 7, 10743.

[277] H. T. Zhang , J. W. Zhang , G. Huang , Z. Y. Du , H. L. Jiang , Chem. Commun. (Camb.) 2014, 50, 12069. 25164253 10.1039/c4cc05571c

[278] X. J. Liu , Y. C. Zhao , F. Li , Biosens. Bioelectron. 2021, 173, 112832. 33234387 10.1016/j.bios.2020.112832

[279] W. J. Dang , Y. M. Sun , H. Jiao , L. Xu , M. Lin , J. Electroanal. Chem. 2020, 856, 113592.

[280] C. S. Liu , C. X. Sun , J. Y. Tian , Z. W. Wang , H. F. Ji , Y. P. Song , S. Zhang , Z. H. Zhang , L. H. He , M. Du , Biosens. Bioelectron. 2017, 91, 804. 28152486 10.1016/j.bios.2017.01.059

[281] Y. Wang , H. L. Ge , G. Q. Ye , H. H. Chen , X. Y. Hu , J. Mater. Chem. B 2015, 3, 3747. 32262849 10.1039/c4tb01869a

[282] Q. X. Wang , Y. Z. Yang , F. Gao , J. C. Ni , Y. H. Zhang , Z. Y. Lin , ACS Appl. Mater. Interfaces 2016, 8, 32477. 27933823 10.1021/acsami.6b11965

[283] J. E. d. S. Souza , G. P. D. Oliveira , J. Y. N. H. Alexandre , J. G. L. Neto , M. B. Sales , P. G. d. S. Junior , A. L. B. d. Oliveira , M. C. M. d. Souza , J. C. S. d. Santos , Electrochem 2022, 3, 89.

[284] K. S. Moreira , L. S. Moura , R. R. C. Monteiro , A. L. B. de Oliveira , C. P. Valle , T. M. Freire , P. B. A. Fechine , M. C. M. de Souza , G. Fernandez‐Lorente , J. M. Guisan , J. C. S. dos Santos , Catalysts 2020, 10, 414.

[285] A. M. da Fonseca , J. C. S. dos Santos , M. C. M. de Souza , M. M. de Oliveira , R. P. Colares , T. L. G. de Lemos , R. Braz , Ind. Crops Prod. 2020, 145, 111890.

[286] J. D. Cui , S. Z. Ren , B. T. Sun , S. R. Jia , Coord. Chem. Rev. 2018, 370, 22.

[287] S. Patra , T. H. Crespo , A. Permyakova , C. Sicard , C. Serre , A. Chausse , N. Steunou , L. Legrand , J. Mater. Chem. B 2015, 3, 8983. 32263029 10.1039/c5tb01412c

[288] W. L. Liu , N. S. Yang , Y. T. Chen , S. Lirio , C. Y. Wu , C. H. Lin , H. Y. Huang , Chem.‐A Eur. J. 2015, 21, 115. 10.1002/chem.201405252 25384625

[289] L. P. Du , W. Chen , P. Zhu , Y. L. Tian , Y. T. Chen , C. S. Wu , Biotechnol. J. 2021, 16, 1900424. 10.1002/biot.201900424 32271998

[290] G. S. Chen , X. X. Kou , S. M. Huang , L. J. Tong , Y. J. Shen , W. S. Zhu , F. Zhu , G. F. Ouyang , Angew. Chem. Int. Ed. 2020, 59, 2867. 10.1002/anie.201913231 31749284

[291] C. E. Wang , K. M. Liao , ACS Appl. Mater. Interfaces 2021, 13, 56752. 34809426 10.1021/acsami.1c13408

[292] V. Lykourinou , Y. Chen , X. S. Wang , L. Meng , T. Hoang , L. J. Ming , R. L. Musselman , S. Q. Ma , J. Am. Chem. Soc. 2011, 133, 10382. 21682253 10.1021/ja2038003

[293] P. Li , S. Y. Moon , M. A. Guelta , S. P. Harvey , J. T. Hupp , O. K. Farha , J. Am. Chem. Soc. 2016, 138, 8052. 27341436 10.1021/jacs.6b03673

[294] N. S. Rios , D. M. A. Neto , J. C. S. dos Santos , P. B. A. Fechine , R. Fernandez‐Lafuente , L. R. B. Goncalves , Int. J. Biol. Macromol. 2019, 134, 936. 31121223 10.1016/j.ijbiomac.2019.05.106

[295] R. M. Bezerra , R. R. C. Monteiro , D. M. A. Neto , F. F. M. da Silva , R. C. M. de Paula , T. L. G. de Lemos , P. B. A. Fechine , M. A. Correa , F. Bohn , L. R. B. Goncalves , J. C. S. dos Santos , Enzyme. Microb. Technol. 2020, 138, 109560. 32527529 10.1016/j.enzmictec.2020.109560

[296] H. L. Bonazza , R. M. Manzo , J. C. S. dos Santos , E. J. Mammarella , Appl. Biochem. Biotechnol. 2018, 184, 182. 28664524 10.1007/s12010-017-2546-9

[297] T. C. de Souza , T. D. Fonseca , J. D. Silva , P. J. M. Lima , C. Neto , R. R. C. Monteiro , M. V. P. Rocha , M. C. de Mattos , J. C. S. dos Santos , L. R. B. Goncalves , Bioprocess Biosyst. Eng. 2020, 43, 2253. 32725440 10.1007/s00449-020-02411-8

[298] K. Liang , R. Ricco , C. M. Doherty , M. J. Styles , S. Bell , N. Kirby , S. Mudie , D. Haylock , A. J. Hill , C. J. Doonan , P. Falcaro , Nat. Commun. 2015, 6, 7240. 26041070 10.1038/ncomms8240 PMC4468859

